# Polymer Composites Reinforced with Residues from Amazonian Agro-Extractivism and Timber Industries: A Sustainable Approach to Enhancing Material Properties and Promoting Bioeconomy

**DOI:** 10.3390/polym16233282

**Published:** 2024-11-25

**Authors:** Odilon Leite-Barbosa, Claúdia Carnaval de Oliveira Pinto, Jôse Maria Leite-da-Silva, Erick Max Mourão Monteiro de Aguiar, Valdir Florencio Veiga-Junior

**Affiliations:** Military Institute of Engineering—IME, Praça General Tibúrcio 80, Urca, Rio de Janeiro 22290-270, Brazil; odilonleitebc@gmail.com (O.L.-B.); ccop1992@gmail.com (C.C.d.O.P.); leite.jose@ime.eb.br (J.M.L.-d.-S.); mourao.erick@hotmail.com (E.M.M.M.d.A.)

**Keywords:** Amazonian residues, lignocellulosic materials, bioactive compounds, polymer composites, timber industry, agro-extractivism industry, sustainability, circular economy, mechanical properties

## Abstract

The Amazon Region (AR), with its vast biodiversity and rich natural resources, presents a unique opportunity for the development of sustainable polymer composites (PCs) reinforced with residues from both timber and agro-extractivism industries. This study explores the potential of Amazonian residues, such as sawdust, wood shavings, and agro-industrial by-products such as açaí seeds and Brazil nut shells, to enhance the mechanical, thermal, and environmental properties of polymer composites. By integrating these natural materials into polymer matrices, significant improvements in the composite performance were achieved, including increased tensile strength, thermal stability, and biodegradability. The study also highlights the environmental and economic benefits of using these residues, promoting waste reduction and supporting a circular economy in the region. Through case studies and detailed analyses, this study demonstrates the feasibility and advantages of incorporating Amazonian residues into composites for a wide range of applications, from construction materials to consumer goods. This approach not only adds value to the by-products of Amazonian industries, but also contributes to the global effort toward sustainable material development.

## 1. Introduction

The Amazon Region (AR), which has vast biodiversity and rich natural resources, supports numerous economic activities, particularly in timber extraction and agro-extractivism industries [1,2]. However, these industries generate significant amounts of waste, primarily in the form of underutilized or discarded residues from wood processing. These residues present not only an environmental challenge but also an opportunity to promote a circular economy [3]. By integrating these Amazonian residues into polymer composites (PCs), the material properties can be enhanced while simultaneously advancing sustainable practices, thereby reducing waste and creating value from what would otherwise be discarded. This approach not only contributes to environmental sustainability but also fosters a circular economy, in which resources are continuously repurposed and reused [4,5,6].

This vast Amazonian region, spanning several South American countries, is a significant producer of various organic residues from timber extraction and agro-extractivism activities, including the harvesting of açaí [7], Brazil nuts (also known as Pará nut or Amazon nut) [8], Maçaranduba [9], and other native products. These processes generate a substantial number of lignocellulosic by-products, which are often underutilized or discarded. These residues, which are rich in cellulose, hemicellulose, and lignin, have enormous potential for incorporation into PCs, where they can enhance the mechanical and thermal properties, while also contributing to the biodegradability of the final material. By effectively utilizing these by-products, not only is waste reduced, but the overall environmental impact is also minimized, supporting sustainable practices and advancing the circular economy [10,11,12].

Several studies have explored the use of agricultural and industrial residues in polymeric composites for different applications, highlighting their potential to enhance material properties and promote environmental sustainability. For example, eucalyptus sawdust [13], oil palm fibers [14], rice husks or straw [15,16], coconut coir [17], sugarcane bagasse [18], and wheat straw fibers [19] have been widely studied for their potential in reinforcing PCs, demonstrating substantial improvements in tensile strength and impact resistance. Given these successful applications of residues in polymeric composites across the globe, similar strategies can be effectively applied to abundant and underutilized residues from the Amazon Region, such as by-products from the extensive timber industry and agro-extractivism sector, to create sustainable and high-performance materials [20,21].

The use of polymeric matrices in composites is crucial because of their ability to bind reinforcement materials, such as natural fibers or industrial residues, into a cohesive structure that exhibits enhanced mechanical, thermal, and chemical properties [22,23]. These polymer matrices can be broadly classified into thermoset and thermoplastic types, each with distinct advantages depending on the intended application. Thermoset matrices, such as epoxy resins, provide excellent thermal stability and resistance to chemical attack but are more brittle and cannot be remolded once cured. In contrast, thermoplastic matrices such as polyethylene (PE) and polypropylene (PP) offer superior impact resistance and the ability to be reshaped with heat, making them highly versatile for various applications. For instance, PE and PP are commonly used in packaging and automotive parts because of their toughness and ease of processing, whereas biodegradable polymers, such as polybutylene adipate-co-terephthalate (PBAT), are favored for sustainable applications [24,25]. The selection of the matrix significantly influences the overall performance of the composite, including its resistance to environmental factors such as moisture absorption, chemical exposure, and elevated temperatures. The incorporation of reinforcement materials into these matrices can lead to significant improvements in the tensile strength, stiffness, impact resistance, and thermal stability, making them suitable for a wide range of applications, from automotive parts to medical devices [26]. Additionally, by using natural or industrial residues as fillers, the environmental impact of polymer composites can be reduced, thus supporting sustainability and the principles of circular economy. This not only adds value to waste materials but also reduces reliance on non-renewable resources, placing PCs as a key focus in the development of next-generation materials [27,28].

This study aimed to investigate the potential of Amazonian residues, particularly those from the timber and agro-extractivism industries, as reinforcement in PCs. By examining the types, sources, and characteristics of these residues, this study aims to identify their suitability for enhancing the mechanical, thermal, and environmental properties of polymer matrices. Furthermore, this study explored the environmental impact and economic viability of using these residues, highlighting their role in promoting a circular economy within the region.

## 2. Amazonian Timber and Agro-Extractivism Bioindustries

### 2.1. General Description of Bioindustry in the Amazon

The history of humankind shows that civilizations have always sought products from nature for various purposes [29]. As new lands are being explored, more natural resources have been discovered [30,31]. When European explorers discovered the Amazon, significant efforts were made to uncover its riches, not only in mineral deposits but also in its flora and fauna. The exploitation of rubber is a historical example of Amazon’s potential, which can be considered the beginning of bioindustry in the region [32]. In the Amazonian context, bioindustry is defined as industries with commercial potential that use natural resources from Amazonian biodiversity in areas such as energy, the environment, agriculture, food, animals, and human health. These industries develop products and processes using biotechnology and bioscience-related activities such as consulting, laboratories, and research at all technological levels [33,34]. In just one state within the Brazilian Amazon, only 44 registered enterprises participate in production, processing, and biotechnological studies, including 40 companies and 4 research institutes. The companies include seven medium-sized, six small-sized, and twenty-seven micro-enterprises that employ approximately 4000 workers and generate BRL 700 million in revenue. These enterprises operate in sectors such as food, biorefinery, bioinputs, cosmetics, environment, bioenergy, pharmaceuticals, chemicals, and innovation, with thirty-four enterprises in the modern third-generation implementation phase [35].

The Amazon forest area comprises nine countries (Bolivia, Colombia, Ecuador, Peru, Venezuela, Suriname, Guyana, French Guiana, and Brazil), with 66% of this area located in the Brazilian territory [36]. Approximately one billion people depend, in part, on native species from nature, including animal meat, edible insects, and plants with traditional healing properties. The Amazon occupies the greatest biodiversity on Earth, accounting for approximately 13% of the species already described in the literature and within approximately 0.5% of the total land area. This biodiversity includes approximately 50,000 plant species, 2.5 million insects, 350 primates, 800 amphibians and reptiles, 1300 birds, and 3000 fish species. Only 1 hectare of the Amazon rainforest contains more tree species than the entire area of Western Europe. These characteristics make the Amazon a field with high biodiversity value [37].

The rich biodiversity of the Amazon is not only reflected in its vast array of wildlife but also in its agricultural output. In the central region of the Amazon, approximately 303 Brazilian cities are involved in the production of Amazonian fruits and seeds, such as açaí, andiroba, Brazil nuts, cumaru, and cupuaçu. Among them, only 39 cities process these raw materials [38]. The use of Amazonian products has stimulated research on the residues of raw materials, turning them into bioinputs for other products, such as composites, and making the cycle of the original raw materials more sustainable. Composites with the addition of bio-based materials become biocomposites, which include fungi found in guarana residues that act as binders for lignocellulosic material substrate particles [39], cassava starch with tururi fiber to produce biopolymers [40], jute fibers with wood residues [41], mango kernels to produce biopolymers [42], and pirarucu fish scales [43]. The potential of Amazonian-derived biocomposites is promising and requires further study.

Researchers need to increasingly study the potential of biocomposites derived from Amazonian bioinputs as sustainable alternatives owing to significant environmental concerns related to plastic disposal [44,45]. Amazonian bioinputs for composites are diverse and can be produced from the raw material itself, by-products, or residues. This diversification is due to the variety of properties and purposes that one seeks to achieve. The primary class of bioinputs is vegetable oils such as copaiba, andiroba, and palm oil. Rodrigues et al. [46] incorporated copaiba oil (*Copaifera* sp.), in both direct and microencapsulated forms, into *Xanthosoma mafaffa* Schott starch-based films, resulting in a biocomposite with higher hydrophobicity, lower tensile strength and elongation, and inhibition of tested Gram-positive bacteria. Silva et al. [47] developed a biomimetic material for wound treatment using poly(e-caprolactone) and andiroba seed oil.

In addition to vegetable oils, several studies have been conducted on composites formed from natural fibers. They have been developed using raw materials produced in AR, particularly sisal [48], jute [49], and malva [50]. However, other fibers that use both raw materials and residues are gaining interest. The latter class contributes to the reduction in the environmental impact of conventional polymers. Rojas-Bringas et al. [51] used Brazil nut husks with starch from three dissimilar sources to produce a biocomposite in which the Brazil nut fibers acted as a natural filler with good ecological quality. Inamura et al. [52] prepared a biocomposite of gelatin with Brazil nut husks, including acrylamide as a copolymer in glycerin, to test the effect of electron beam irradiation, observing that crystallinity decreased with increased irradiation. Curauá is a plant that has been extensively tested as an alternative natural fiber. Souza et al. [53] investigated the autogenous self-healing capacity of a curauá fiber-reinforced cement biocomposite, with high self-healing capacity. Castro et al. [54] developed a biocomposite with curauá fiber, hydroxylated polybutadiene, and high-density biopolyethylene, where the curauá fiber improved the mechanical properties of the biocomposite. Frollini et al. [55] performed mechanical and water absorption tests, and a morphology study of a biocomposite using poly (butylene succinate) (PBS) as the polymer matrix and curauá fiber molded by compression, yielding better results than using the individual components.

### 2.2. Production and Economic Impact

Despite the vast biological diversity of the region, wood production in the Brazilian Amazon is predominantly concentrated on a small group of commercially valuable species. Between 2007 and 2020, the 20 most exploited species accounted for approximately 52% of the total volume of roundwood, amounting to approximately 81 million cubic meters [56]. Among these, Maçaranduba (*Manilkara huberi*) is the most exploited species [57,58], with an accumulated volume of approximately 11.2 million cubic meters, corresponding to approximately 7.2% of the total volume produced during this period. Following Maçaranduba, Cedrinho (*Erisma uncinatum*) [59] and Cupiúba (*Goupia glabra*) [60] were also widely exploited, with accumulated volumes of approximately 8.9 million and 8.6 million cubic meters, respectively, representing about 5.8% and 5.6% of the total production. The average annual production of roundwood in the Brazilian Amazon has varied between ten million cubic meters and 13 million cubic meters [56,61].

The average annual production of roundwood in the Brazilian Amazon has varied significantly, from 14.9 million cubic meters in 2012 to 11.2 million cubic meters in 2023, according to data from the Brazilian Institute of Geography and Statistics (IBGE) [62]. This trend reflects a gradual decline over time, influenced by the economic, environmental, and regulatory factors affecting the timber industry. Consequently, residue generation, including sawdust and wood shavings, has also followed this trend, decreasing from 8.2 million cubic meters in 2012 to 6.2 million cubic meters in 2023. These residues constitute up to 55% of the total processed wood, representing a substantial volume of underutilized biomass [63]. For instance, in years when total wood production reached approximately 12 million cubic meters, approximately 6.6 million cubic meters of residue was generated [62,63]. The consistent proportion of residues highlights a stable potential for reuse in polymer chemistry. This significant volume of by-products highlights the potential environmental impact of timber processing if the residues are not adequately managed or repurposed. In addition, the economic impact of these residues should not be underestimated. The integration of sawdust and other wood residues into polymer composites not only adds value to these by-products but also represents a significant opportunity for bioeconomy. The use of timber industry residues contributes to both environmental sustainability and economic growth by reducing waste and creating high-performance sustainable materials that can be used in various applications from construction to consumer goods. This approach aligns with the principles of the circular economy and positions Amazon as a leader in sustainable material innovation.

In addition to the timber industry, other agro-extractivism industries serve a fundamental role in the Amazon economy, significantly contributing to the supply of residues that can be used for the development of PCs. Among these industries, non-timber forest products, such as açaí [64]; Brazil nuts [65]; and oils, such as copaiba and andiroba [66], stand out. The annual production of açaí in Brazil has grown significantly in recent years, reflecting increasing demand in both domestic and international markets. According to data from the Brazilian Institute of Geography and Statistics (IBGE), in 2022, Brazil produced approximately 1.7 million tons of açaí fruits, generating a revenue of BRL 6.17 billion (more than USD 1 billion) [62,67]. This growth is driven by the expansion of cultivation areas and the intensification of palm management, especially in the main producing regions of the Eastern Amazon, which continue to be the largest producing areas in the country.

Another highly exploited activity in the Amazon is the extraction of Brazil nuts, which are currently called “Amazon nuts”. The annual production of this nut is a vital economic activity for many communities in the Amazon, as well as generating lignocellulosic residues that present exciting potential for the development of reinforced PCs [68]. Bolivia remains the top producer of Brazil nuts, accounting for 79% of the global production in the 2022/23 season, with a production of 22,000 metric tons. According to data from the International Nut and Dried Fruit Council, Peru and Brazil contributed 16% (4, 500 metric tons) and 5% (1, 500 metric tons), respectively [69]. These countries, all located within the Amazon rainforest, not only produce significant quantities of Brazil nuts but also generate substantial residues. These residues offer valuable opportunities for sustainable applications such as their incorporation into PCs. In addition to Brazil nuts and açaí, whose residues have been studied for their application in composites, the extractive industry in the Amazon encompasses a wide range of natural products that are fundamental to the local and national economy. In 2022, other notable Amazon non-timber forest products included babassu (almond), with a production of 148,635 tons; erva-mate, 35,964 tons; pequi (fruit), 3657 tons; and urucum (seed), 989 tons [62].

**Figure 1:**
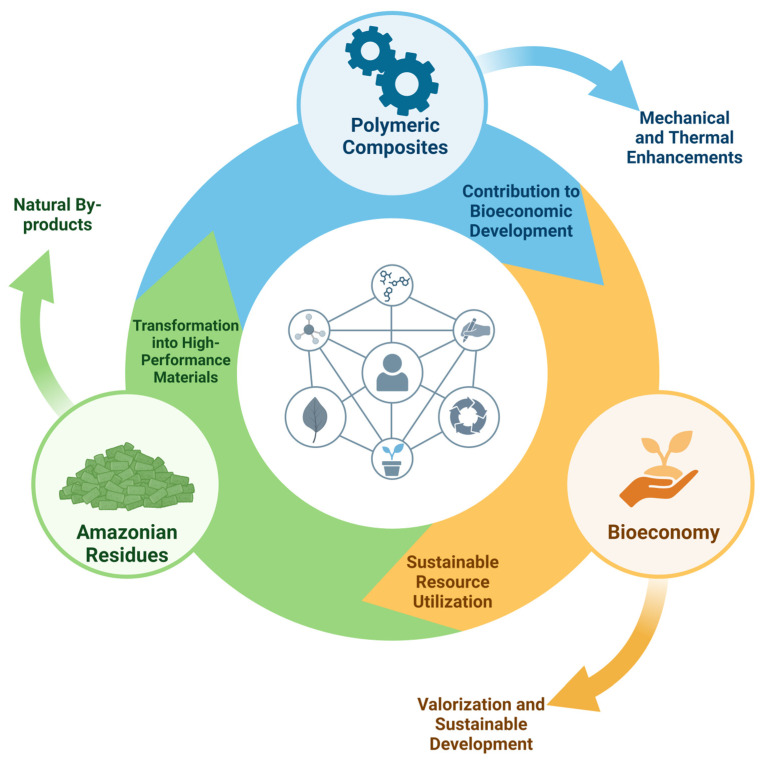
Interconnections between Amazonian residues, polymeric composites, and bioeconomy. The central portion represents the role of human activities in sustainable development, with icons symbolizing key elements, such as chemical interactions, sustainable resource management, material reuse, and the creation of innovative materials.

The interconnected relationship between these diverse agro-extractivism activities and the development of PCs can be visually represented through the conceptual model depicted in Figure 1. This model illustrates how Amazonian residues, such as those from Brazil nuts, açaí, and other natural products, can be integrated into polymeric matrices to produce high-performance materials. In turn, these composites contribute to a broader bioeconomy by offering sustainable and economically valuable alternatives to traditional materials. The central portion of Figure 1 illustrates the interconnectedness of key elements involved in the development of PCs using Amazonian residues. At the core, the human figure symbolizes the role of human activities, such as research, innovation, and development, in the sustainable use of natural by-products. The icons surrounding this central figure represent various critical components: chemical processes (molecular structure icon), sustainable waste management (recycling icon), valorization of natural resources (leaf icon), and transformation of these resources into high-performance materials (plant growth icon). These interconnected elements emphasize the cyclical relationship between agro-extractivism, material development, and bioeconomic advancement.

In addition to its environmental impact, the Amazon bioindustry is a significant source of employment and income for local communities. The production and processing of products, such as açaí and Brazil nuts, employ thousands of workers, often in regions where economic opportunities are limited. The export of these products to international markets, such as Europe and the United States, also contributes substantially to the local economy [70]. In 2022, the production of copaiba oil in Brazil reached 265 tons, generating a production value of approximately 10.188 million reais, demonstrating the economic importance of these products [62]. However, bioindustry faces significant challenges in terms of sustainability and efficient waste management. The lack of adequate infrastructure and the need for more advanced technologies to process and reuse by-products are barriers that must be overcome. Nonetheless, with the increasing demand for sustainable products, there is a clear opportunity to expand the use of residues in PCs, as illustrated in Figure 1, promoting the bioeconomy in the region and enhancing the economic and environmental benefits of the Amazon bioindustry [70,71].

### 2.3. Impact of Chemical Composition on Polymeric Composite Properties

The chemical composition of lignocellulosic residues, such as seeds, shells, and fibers, directly influences the performance of PCs in various ways. The high cellulose and hemicellulose contents in the residues enhance the stiffness and mechanical strength of the composites because these components strongly interact with the polymer matrix, promoting a more homogeneous dispersion of the reinforcement [72,73]. However, the presence of lignin and extractives can have mixed effects. While lignin can improve thermal stability and barrier properties, excessive amounts may hinder the adhesion between the matrix and residues, reducing the cohesion of the composite [74]. Similarly, extractives, including phenolic compounds and fatty acids, can affect the interfacial properties [75].

Residues rich in nonpolar extractives, such as those found in certain seeds and shells, can reduce chemical interactions with polar polymers, resulting in composites with low cohesion and structural strength. In contrast, residues with higher levels of polar components, such as cellulose and hemicellulose, tend to exhibit better compatibility with hydrophilic polymer matrices, thereby enhancing the overall performance of the material [76,77].

In addition to lignocellulosic materials, other components found in residues from agro-extractive practices also contribute to specific PCs’ properties. Acai seeds (Euterpe oleracea) are another valuable by-product that can be effectively utilized in PCs. Murillo-Franco et al. [78] reported that acai seeds contain 11.58% cellulose, 37.88% hemicellulose, and 14.12% lignin, whereas Buratto et al. [79] reported slightly different values of 8.5%, 48.1%, and 16.4%, respectively. These components are crucial for the mechanical properties of composites, with hemicellulose playing a significant role in improving flexibility and lignin contributing to increased stiffness and thermal stability. Additionally, the presence of extractives (water-soluble at 4.09% and ethanol-soluble at 11.76% in Murillo-Franco et al. [78]) and fats (2.64–3.5%) can affect the interaction of the matrix with the reinforcement, further enhancing the performance of the composite. An interesting aspect of açaí is its high mannan content, which has been highlighted in several recent studies, such as that of Monteiro et al. [80], who investigated the high concentration and yield of mannose from açaí seeds via mannanase-catalyzed hydrolysis. For example, Rambo et al. [81] reported that mannan accounts for approximately 53.8% of the carbohydrates in seeds. This high mannan content, which can have a crystalline structure, potentially contributes to the mechanical properties of composites by enhancing their dimensional stability and strength, although further studies in the field of materials science are still required [82,83].

Studies on palm kernel shell (PKS), a residue from the palm oil industry, have revealed its potential for use in PCs. Fuadi et al. [84] and Okoroigwe et al. [85] found that PKS contains a high lignin content, ranging from 53.40% to 53.85%, which enhances thermal stability but may decrease compatibility with polar matrices, leading to reduced mechanical strength. Additionally, cellulose (up to 29.70%) and hemicellulose (26.11% to 47.70%) contents provide reinforcement but can increase water absorption, affecting dimensional stability. Porosity (up to 28%) can aid in better matrix impregnation and improve mechanical interlocking, although it may also contribute to brittleness [86]. This composition highlights PKS as a promising yet challenging material for composite applications. Similarly, palm kernel cake, another by-product of the palm oil extraction process, has a high content of oily compounds, such as triglycerides and fatty acids [87]. These oily components can function as natural plasticizers within the polymer matrix, thereby improving the flexibility and impact resistance of the composite [88].

Furthermore, sawdust from *Manilkara huberi* shows potential for PCs because of its 69.41% holocellulose and 34.68% lignin contents [89]. High holocellulose enhances strength and stiffness, whereas lignin improves thermal stability [71,72,73]. Overall, Maçaranduba sawdust could improve both mechanical and thermal properties in composites.

In summary, the chemical makeup of Amazonian timber and agro-extractivism residues directly affect the performance of PCs. In addition to the well-established effects of cellulose, hemicellulose, and lignin, compounds such as mannan, oils, and phenolic compounds also play significant roles in enhancing the mechanical, thermal, and chemical properties of composites. A better understanding of the composition of these materials, as detailed in Table 1, is essential for optimizing the design of high-performance sustainable composites.

In addition to cellulose, hemicellulose, and lignin, other components present in lignocellulosic residues play crucial roles in enhancing the properties of PCs. Extractives, such as phenolic compounds and fatty acids, can act as natural plasticizers or antioxidants, improving flexibility and durability [74]. Additionally, components such as waxes and proteins found in some seeds and shells can improve the interfacial adhesion between the polymer matrix and reinforcement, leading to stronger, more cohesive composites [96,97]. However, studies focusing specifically on the role of these components in PCs are limited, highlighting the need for further research to fully understand and optimize their potential contributions to composite performance.

## 3. Types of Polymer Matrices Applicable in Composites with Residues

In the development of PCs reinforced with Amazonian agro-extractivism and timber-industry residues, the choice of the polymer matrix is crucial for the performance and sustainability of the final material [98,99]. This is because the polymer matrix not only binds the reinforcement materials together, providing structural integrity, but also significantly influences the mechanical properties, thermal stability, and environmental impact of the composite [100]. For instance, thermoplastics offer recyclability and ease of processing, which are advantageous for applications that require durability and environmental responsibility [101]. Thermosets, however, provide superior thermal and chemical resistance, making them suitable for high-performance applications, although they are more challenging to recycle [102]. Biodegradable polymers enhance sustainability by being compostable and reducing long-term environmental impact [103]. Thus, each type of polymer matrix presents unique benefits that must be carefully matched to the specific requirements of the intended application [104].

**Figure 2:**
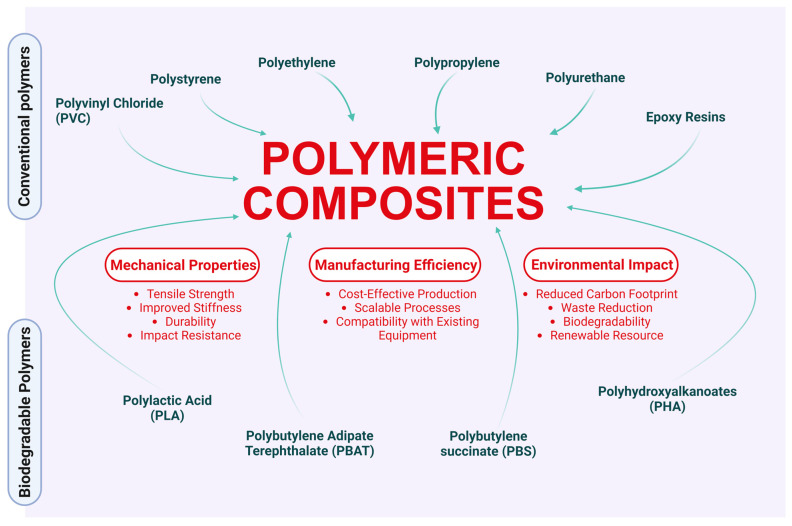
Contribution of conventional and biodegradable polymers to polymer composites.

Figure 2 illustrates how conventional and biodegradable polymers contribute to the formation of PCs, highlighting the impact of different types of polymers on the mechanical properties, manufacturing efficiency, and environmental impact of the composites. Conventional polymers, including PP, PE, and epoxy resins, are often selected for their durability, robustness, and cost-effectiveness in various industrial applications. However, with the increasing demand for sustainable materials, biodegradable alternatives such as polylactic acid (PLA) and PBAT have become attractive because of their ability to degrade under specific environmental conditions [105]. Studies have shown that PP and PE can decompose in landfills over hundreds of years. For instance, PP exhibits high resistance to degradation, and even after five years in a landfill, it only shows initial signs of deterioration, such as the formation of microplastics and biofilms of microorganisms on the material surface [106]. In terms of the environmental impact, the production of 1 kg of PP emits approximately 1.58 kg of CO_2_ equivalent, highlighting a considerable carbon footprint [107]. These factors underscore the complexity of managing conventional plastic waste.

However, biodegradable polymers, such as PLA and PBAT, offer a more sustainable solution. Danyluk et al. [108], for example, observed that PLA bottles without caps and labels were completely composted after four weeks in an industrial composting environment. The study also documented the complete degradation of PLA bottles with HDPE and PP caps and labels by the eighth week, with only small residues found inside the caps and labels, which acted as barriers, limiting microbial access to PLA. By week 12, no significant PLA residue was found in the samples, indicating that the material was nearly fully decomposed.

However, in terms of costs, conventional polymers, such as PP, are significantly cheaper than biodegradable polymers. As highlighted in the European Commission’s report on the sugar platform for biofuels and biochemicals, PP, for example, costs approximately BRL1500 per ton, while polyhydroxybutyrate (PHB), a biodegradable polymer, can cost up to BRL 6500 per ton, making it four to five times more expensive than PP. PLA has an average cost from BRL 2000 to BRL 2200 per ton, which is still more affordable than PHB but approximately 30% more expensive than PP. PS and PE also have lower costs than biodegradable polymers do. The price of PS is approximately BRL 2100 per ton, while PE, depending on the grade, costs about BRL 1500 per ton. In comparison, PLA, priced between BRL 2000 and BRL 2200 per ton, is more expensive. PHB, at up to BRL 6500 per ton, is significantly more expensive than PS and PE, being approximately three-to-four times the cost of PS and up to five times the cost of PE [109].

Other aspects are also important. Conventional polymers such as PP exhibit high mechanical strength, with studies reporting tensile strength of up to 38 MPa [110], as well as high impact and fatigue resistance, making them ideal for durable structural applications. High-density polyethylene (HDPE) has a tensile strength of up to 31 MPa [111]. However, as mentioned earlier, both PP and HDPE have a low biodegradability rate [106]. However, biodegradable polymers have highly variable characteristics. For instance, PLA exhibits good tensile strength but low elongation at break. According to the findings of Gigante et al. [112], pure PLA exhibits an elastic modulus of 3.04 GPa, a yield strength of 63.6 MPa, and an elongation at break of only 7.1%, making it relatively stiff and brittle. In contrast, PBAT is a more flexible polymer. In addition, Tsou et al. [113], PBAT exhibited a tensile strength of 30 MPa, yield strength of 13 MPa, and elongation at break of 1200%, indicating high ductility. Therefore, although PLA tends to be more brittle, PBAT enhances the elasticity of the blends. Technological advances, such as the modification of polymer matrices and addition of reinforcing fillers, have improved these mechanical properties, making biopolymers more competitive in engineering applications. Thus, the choice between these types of polymers involves balancing the technical performance and environmental impact, with biodegradable polymers becoming increasingly viable in applications that require sustainable solutions.

Thermoplastic polymers are widely used in composite formulations because of their ability to repeatedly melt and reshape, offering significant processing flexibility and adaptability for various applications [114]. These polymers are favored owing to their excellent chemical resistance and mechanical robustness, making them ideal candidates for integrating natural fillers [115,116]. In the context of composites reinforced with Amazonian residues, thermoplastics, such as PP and PE, are particularly effective because they can be processed at lower temperatures and molded into complex shapes, allowing for the efficient incorporation of natural materials, such as wood fibers, seeds, and shells [117]. This adaptability not only enhances the mechanical properties and thermal stability of the resulting composites but also aligns with sustainable practices by utilizing abundant and underutilized natural resources [118].

Polypropylene has been extensively studied along with natural fillers, such as wood fibers and agricultural residues. The incorporation of these fillers enhances the mechanical properties of the composite, particularly its tensile strength and stiffness, while also contributing to environmental sustainability by reducing its dependency on virgin polymer materials [119]. Similarly, HDPE, which is often derived from post-consumer recycled sources, bolsters the sustainability profile of composites when combined with Amazonian lignocellulosic residues. This constructive interaction not only enhances the performance characteristics of the material but also aligns with the principles of a circular economy, supporting the efficient use of natural resources within the region [120]. In addition to PP and HDPE, other thermoplastics, such as polystyrene (PS) and polyvinyl chloride (PVC), can also be used in composites reinforced with natural waste.

Several studies have shown that the incorporation of natural residues into polymer matrices, such as PE, results in significant improvements in the mechanical properties of composites compared to those of pure polymers. Campos et al. [121] used Brazil nut pod fibers as reinforcement in HDPE composites and observed a substantial increase in the tensile strength and flexural modulus of the composites compared to pure HDPE, with up to a 20% increase in tensile strength and even greater improvement in the flexural modulus. On the other hand, Souza et al. [122] investigated the use of textile fiber residues in HDPE composites and found impressive increases of 53.5% in tensile strength and 112.5% in the Young’s modulus (elastic modulus) when 10% fibers were added to the composite compared to pure HDPE. Additionally, Gomes et al. [123] studied recycled low-density polyethylene (LDPE) composites reinforced with jupati particles, observing a 17.9% increase in the Young’s modulus with the addition of 15% jupati particles; however, the modulus of rupture showed a decrease.

Thermosetting polymers are characterized by their irreversible curing process, which results in materials with high rigidity, chemical resistance, and thermal stability. These properties make thermosets ideal for applications requiring structural integrity and long-term durability [102]. When reinforced with Amazonian residues, such as wood shavings and bark, thermosetting matrices can produce composites that not only exhibit superior mechanical properties but also contribute to waste valorization by utilizing by-products that would otherwise be discarded [116,124]. The integration of these natural fillers can enhance the mechanical strength and stiffness of the composites, making them more robust while promoting sustainable material design [125]. Among the most used thermosetting polymers are epoxy resins, unsaturated polyester resins, and phenolic resins, each offering distinct advantages depending on the application.

Epoxy resins, like other thermosets, have been extensively studied in the context of PCs. Epoxy resins are known for their strong adhesive properties and compatibility with various reinforcement materials [126]. The incorporation of Amazonian residues into epoxy matrices can lead to the development of high-performance composites suitable for various industrial applications, from construction to automotive components. For example, Mendes et al. [127] used sawdust from a non-Amazonian species as reinforcement in epoxy composites, resulting in an approximately 43.5% increase in tensile strength compared to pure epoxy resin, with the addition of 10% by weight of sawdust to the matrix. Although this study used sawdust from a non-Amazonian species, the approach could be similarly applied to residues from the Amazon timber industry, such as local wood sawdust.

In line with the growing demand for environmentally friendly materials, biodegradable polymers like PLA and PBAT have gained significant attention in the development of sustainable composites. PLA is derived from renewable resources, such as corn starch or sugarcane, and is capable of degrading under specific environmental conditions, making it an attractive choice for applications where environmental impact is a primary concern [128]. On the other hand, PBAT, although fossil-based, is biodegradable and can break down more rapidly in natural environments compared to conventional plastics [129,130].

To address these challenges, current research has increasingly focused on developing 100% eco-sustainable composites. This involves replacing nonbiodegradable polymers with biodegradable alternatives derived from renewable resources. By integrating Amazonian agro-extractivism residues with these biodegradable matrices, it is possible to create composites that not only retain desirable mechanical properties but also enhance the biodegradability of the final product. For example, Ferreira et al. [131] investigated biodegradable composites based on PBAT reinforced with three different natural fibers from the Amazon forest: *Croton lanjouwensis*, *Malvastrum tomentosum*, and *Trema micrantha*. The study with these species showed that all composites presented a significant increase in Young’s modulus compared to pure PBAT, ranging from 48% to 72%. In another study, Pinheiro et al. [132] explored PBAT composites reinforced with fibers from Munguba (*Pseudobombax munguba*), a tree abundant in the flooded areas of the Amazon. The addition of Munguba fibers resulted in a significant increase in the Young’s modulus of the composites, from 51.0 MPa in pure PBAT to up to 97.0 MPa in composites with 20% fibers, representing an approximately 90% increase. However, it was observed that the addition of fibers reduced the tensile strength and elongation at break due to the weak interaction between the fibers and the polymer matrix.

The key characteristics of conventional and biodegradable polymers, including their mechanical properties, manufacturing efficiency, and environmental impact, are summarized in Table 2.

## 4. Polymer Composites with Amazonian Timber Industry Residues

### 4.1. Types and Sources of Timber Residues

The diversity of exploited species is reflected in the variety of available residues, including sawdust, shavings, chips, and other by-products of wood processing [164]. Among the most exploited species in the Amazon, Maçaranduba is known for its dense and durable wood, which generates significant quantities of sawdust during processing [56]. Other widely exploited species include Cedrinho (*Erisma uncinatum*), Cupiúba (*Goupia glabra*), and Jatobá (*Hymenaea courbaril*), each contributing specific residues that can be valorized in composite manufacturing [56,165]. For instance, Maçaranduba sawdust is an abundant and underutilized by-product that can be incorporated into polymer matrices to enhance their mechanical and thermal properties. Similarly, residues from Cupiúba and Jatobá possess characteristics that make them promising candidates to produce reinforced composites. Using these residues not only contributes to environmental sustainability by reducing waste and promoting material recycling but also offers economic benefits by adding value to timber industry by-products [166,167].

Another significant residue is wood shavings, also known as planing chips, which are produced during the planing of wood in sawmills or carpentry shops. Shavings are coarser and longer than sawdust and can be used as fillers in composites. Firewood, including bark, trimmings, and offcuts, is a typical large-volume residue generated during log cutting and planning [168,169]. Additionally, bark removed during preparation of logs constitutes another significant type of residue. Often considered of low value, bark is rich in organic compounds and can be used for energy generation or as raw material in wood-panel production [170]. Proper classification of these residues is essential for optimizing their use in composite manufacturing, ensuring better material valorization and minimizing waste [171].

**Figure 3:**
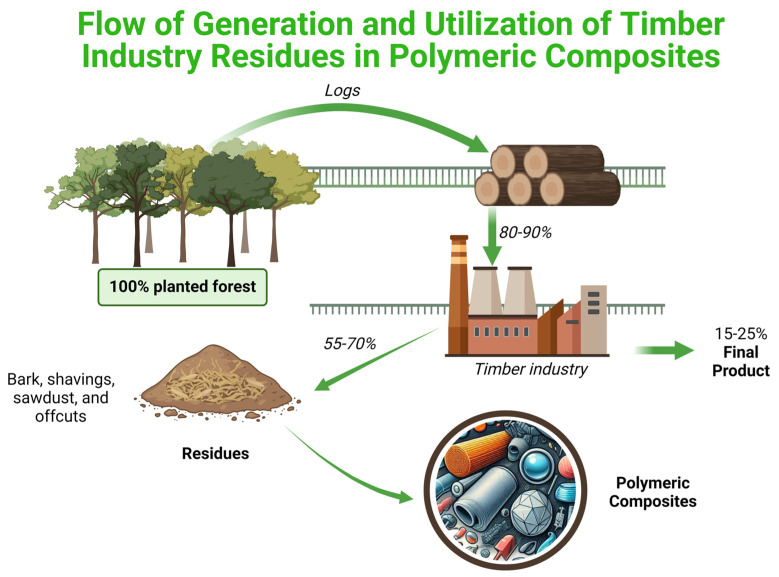
Flowchart illustrating the production process in the wood industry, from the cultivation of planted forests to the generation of residues such as bark, shavings, sawdust, and offcuts.

Figure 3 illustrates the industrial wood-processing chain, where only 15–25% of the original volume of processed logs results in a final product, while the remainder is converted into waste such as bark, shavings, sawdust, and offcuts [172]. These residues, which would normally be discarded, can be reintegrated into the production chain by incorporating them into polymer matrices, adding value by transforming by-products into useful materials for the fabrication of PCs.

Beyond the types of residues already mentioned, it is essential to consider the chemical composition and specific properties of these materials to determine their potential use in PCs [173]. Sawdust, for example, is not only a voluminous residue but also a rich source of cellulose, hemicellulose, and lignin. These chemical components confer high mechanical strength and good thermal stability to sawdust, making it ideal for producing reinforced composites [127]. The cellulose present in materials such as sawdust also contributes significantly to the mechanical properties of composites, enhancing their tensile strength and stiffness, especially when combined with appropriate chemical modifications [174].

As an illustration, Nobre et al. [89] highlighted that sawdust from Maçaranduba is particularly noteworthy for its high lignin content (34.68%) and holocellulose levels (69.41%), which significantly enhance the mechanical performance of composites by providing increased rigidity and thermal stability. Similarly, their study found that other Amazonian woods, like Cedrinho (*Erisma uncinatum*) and Jatobá, exhibit lignin content ranging from 16 to 24% and holocellulose content from 65 to 82%, making them promising candidates for enhancing the structural properties of PCs. Bimestre et al. [94], in a comprehensive study of Amazonian woods, found that Amescla (*Trattinnickia* sp.) exhibited the highest lignin content (33.86%), while Mandioqueira (*Ruizterania albiflora*) had the highest cellulose content (55.90%). Likewise, Mamica de Porca (*Fagara* sp.) was noted for its high hemicellulose content (17.00%). These findings underscore the suitability of these species for composite applications because of their robust chemical compositions.

### 4.2. Residue Availability and Environmental Impact of Timber Industry

The availability of waste generated by the timber industry in the Amazon varies considerably, depending on the locality and the wood species being processed. It is estimated that thirty million tons of wood waste is generated annually in Brazil, of which 91% come from sawmills and lumber industries [175,176]. According to the Document of Forest Origin report by IBAMA (Brazilian Institute of Environment and Natural Resources) in 2017, approximately 938,000 m^3^ of wood residues was produced in the Amazon (northern) region of Brazil alone. The timber industry in the Amazon continues to generate large volumes of waste [61]. Figueira et al. [63] reported that, in 2020, local sawmills processed about 69,300 m^3^ of wood, achieving a production yield of 55%, which left a significant amount of waste with no specific destination. Moreover, Mendoza et al. [168] revealed that about 50% of the volume of logs processed is considered waste, raising environmental concerns due to the lack of alternatives for utilizing these residues. Numerous studies have explored the percentages of waste generated by sawmill companies, highlighting significant variations in the volumes of waste produced. According to Ramos et al. [167], in a key urban area of the Eastern Amazon, companies generate an average of 398.9 m^3^ per month of timber waste, with a total of 12.3 thousand cubic meters per month produced by the thirty-one companies evaluated. The amount of waste generated varies between 0.63 and 919 m^3^ per month, influenced by the consumption of raw materials and the production of each company.

**Figure 4:**
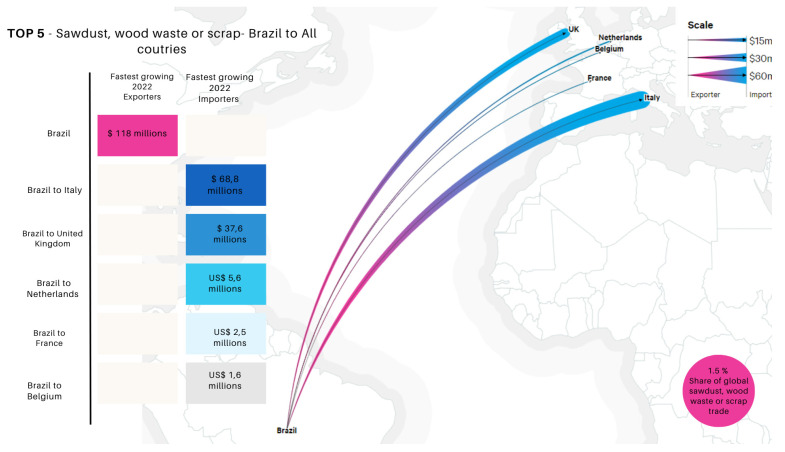
Main export and import routes of sawdust and wood residues from Brazil (2017–2022).

As illustrated in Figure 4, which presents the main export and import routes of sawdust and wood residues from Brazil in 2022, several countries stand out for their significant growth in this trade. According to COMTRADE (United Nations Commodity Trade Statistics Database) [177], Brazil plays a substantial role in the global trade of sawdust and wood residues, contributing approximately 1.5% to this market, translating to a total trade flow of USD 118 million. These residues are directed to multiple destinations, with the top five importing countries demonstrating significant demand. The leading importing country is Italy, with a substantial value of USD 68.8 million. The United Kingdom follows with imports valued at USD 37.6 million, reflecting a strong demand for wood residues. In third place, the Netherlands imports USD 5.6 million, highlighting the diversity of the European market. France, in fourth place, acquires USD 2.5 million, representing a significant fraction of the trade, while Belgium, in fifth place, imports USD 1.6 million, contributing to the commercial dynamics in the region.

**Figure 5:**
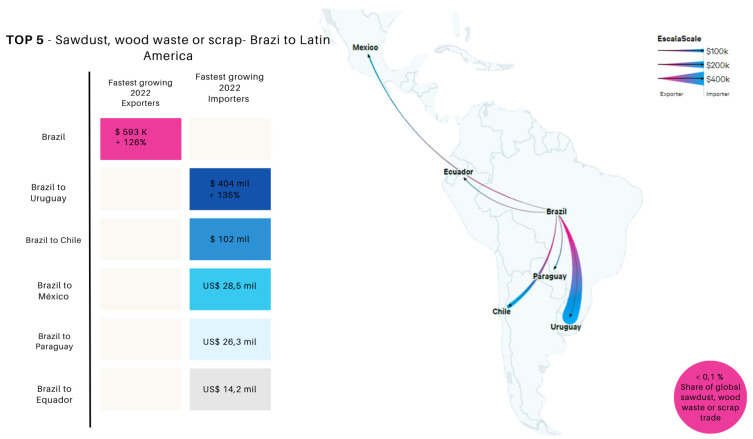
Main export and import routes of sawdust and wood residues from Brazil (2017–2022).

When analyzing the trade flows of Brazilian exports to Latin America (Figure 5), it is observed that Brazil also exports wood residues to neighboring countries. The following data illustrate this dynamic within the top five countries: Uruguay imported USD 404,000 worth of wood residues, showing a notable growth of 135% from 2017 to 2022. Brazil, with a share of less than 0.1% in the global trade of sawdust and wood residues, registered a total export value of USD 593,000, evidencing a growth of 126% over the same period. The top five importing countries of these products were Chile, with an import value of USD 102,000, indicating a developing potential market; Mexico, in third place, with USD 28,500; Paraguay, in fourth place, with USD 26,300; and Ecuador, in fifth place, with USD 14,200 worth of sawdust and wood residues [177]. However, while Brazil is exporting wood residues, such as sawdust, at significant values, there is untapped potential to add even more value to these residues. Instead of merely exporting the raw material, these residues could be utilized domestically to produce high value-added products, such as advanced polymeric composites. This would not only increase the economic value generated from these residues but also contribute to the development of a more sustainable and circular economy in the AR.

### 4.3. Applications of Residues from Amazon Timber Industry in Polymer Composites

Numerous studies in the literature have explored the application of Amazonian timber industry residues in PCs, highlighting their potential to enhance the mechanical, thermal, and environmental properties of these materials [178,179]. Researchers have investigated a wide range of wood residues, such as sawdust, shavings, and bark, incorporating them into various polymer matrices to create composites with improved performance and sustainability [180,181]. A summary of key examples from the literature, illustrating the significant improvements in mechanical properties, such as increased tensile strength and Young’s modulus, is provided in Table 3. While this table offers selected examples, it reflects broader trends observed across multiple studies [182].

Santos et al. [179] investigated the impact of moisture content in mercerized-wood residues on the rupture modulus of thermopressed polyurethane-based composites. The research focused on tropical-wood residues from the Amazon, specifically Louro Itaúba, Louro Gamela, and Maçaranduba, investigating the effects of different NaOH concentrations (5% and 10%) and moisture levels (3% and 12%) during the thermopressing process. The study evaluated residues from Louro Itaúba, Louro Gamela, and Maçaranduba, showing notable increases in the rupture modulus of the composites. For Louro Gamela, the rupture modulus increased from 12 MPa to 16 MPa, while for Louro Itaúba, it increased from 2 MPa to 9 MPa. In the case of Maçaranduba, the rupture modulus rose from 3 MPa to 11 MPa. These results indicate that the incorporation of Amazonian-wood residues enhances the strength of the composites, improving their rupture resistance and making them more suitable for various industrial applications. The study highlights the potential of these residues to improve the mechanical properties of composites while promoting a sustainable solution for the use of by-products from the Amazonian timber industry.

The development of biodegradable composites using natural fiber residues as reinforcement faces challenges due to incompatible interfaces between fillers and matrices. Rocha et al. [183] studied the use of a starch coating as a natural coupling agent to improve the compatibility between lignocellulosic residues, including sugarcane bagasse, Maçaranduba, and Pinus, and a PLA matrix. Table 3 shows that the starch coating significantly improved the stiffness and strength of the composites reinforced with Maçaranduba and Pinus residues. The PLA–Pinus composite exhibited the greatest increase in Young’s modulus, with a 34% increment (from 2.6 GPa to 3.5 GPa), while maintaining almost the same tensile strength, whereas the PLA–Maçaranduba composite showed a 15% increase in Young’s modulus (from 2.6 GPa to 3.0 GPa). On the other hand, the sugarcane bagasse demonstrated less efficiency in the starch coating, with a reduction in Young’s modulus to 2.1 GPa, suggesting that the larger surface area of the bagasse hindered the formation of a uniform coating. In terms of impact absorption, the PLA–Maçaranduba composite also stood out, with a significant increase from 0.193 J/m to 0.357 J/m. Additionally, the thermal properties of the composites were evaluated, showing that the initial decomposition temperature (T10%) of all composites was slightly lower than that of pure PLA, with a reduction of about 10 °C, which is associated with the lower thermal stability of the lignocellulosic residues and starch compared to PLA.

Surdi et al. [180] investigated the effective use of wood residues from the mechanical processing of Amazonian species for high-density particleboard production, offering a sustainable solution to waste-management challenges in the timber industry. The study used residues from *Caryocar villosum*, *Hymenolobium excelsum*, *Mezilaurus lindaviana*, *Erisma uncinatum*, *Tachigali myrmecophyla*, and *Qualea paraensis* to manufacture panels with a nominal density of 850 kg/m^3^ and a thickness of 15.7 mm, utilizing 8% phenol-formaldehyde adhesive. The results showed that particleboards produced with residues from *Caryocar villosum*, *Hymenolobium excelsum*, and *Tachigali myrmecophyla* exhibited superior mechanical properties compared to the others, meeting or exceeding the minimum requirements of the ANSI A208.1 standard for high-density panels (H-1 classification) and floor production (PBU classification). Specifically, panels made with *Caryocar villosum* residues had a rupture modulus of 9.39 MPa and a Young’s modulus of 1616.74 MPa, while panels with *Tachigali myrmecophyla* residues showed the highest rupture modulus at 10.04 MPa. Additionally, the panels made with *Hymenolobium excelsum* exhibited the highest internal bonding strength at 1.41 MPa, making them the best option for structural applications. These findings highlight the potential of transforming wood-processing residues into high-value engineered materials, contributing to resource efficiency and environmental sustainability within the timber industry.

The utilization of jatobá-wood residues combined with short malva fibers in PCs presents an innovative approach to material recycling. As Costa et al. [184] demonstrated, the mechanical properties of these composites highlight the influence of fiber length on tensile strength. The study found that composites reinforced with 15 mm malva fibers achieved a tensile strength of 25.09 MPa, while hybrid composites with a 75/25 ratio of 15 mm fibers and jatobá-wood residues exhibited a slightly higher tensile strength of 26.06 MPa. The microstructural characterization indicated that the incorporation of jatobá-wood residues did not significantly affect the tensile strength, but the presence of fewer voids and the good rigidity in the hybrid composites contributed to their performance. These results suggest that the inclusion of a small percentage of wood residues can be a viable strategy for developing high-performance hybrid composites while promoting the reuse of wood waste.

Another study by Ferreira et al. [185] evaluated jatobá-wood powder as a reinforcement in PP composites, focusing on the influence of PP viscosity on thermal, mechanical, thermomechanical, and morphological properties. The addition of jatobá powder improved the mechanical performance of the composites, particularly in terms of elastic modulus, with an increase of 59% for composites with 40% jatobá powder in the PP H103 matrix (rising from 800 MPa to approximately 2000 MPa) and 50% in the PP H503 matrix. Thermal properties were also enhanced, as the composites exhibited increased heat deflection temperature, with the highest value recorded at 138 °C for PP H103 with 40% jatobá powder compared to 89 °C for neat PP H503. Vicat softening temperature and Shore D hardness also improved with higher filler content, indicating better thermomechanical stability. However, there was a slight reduction in tensile strength (up to 23% lower for PP H103 with 40% jatobá powder) and elongation at break, likely due to poor interfacial adhesion and wood-powder agglomeration observed in the SEM images. Despite these decreases, the study confirmed that jatobá powder enhances the overall stiffness and thermal resistance of the PP composites, making them suitable for applications requiring high-dimensional stability at elevated temperatures.

## 5. Polymer Composites with Agro-Extractivism Industry Residues from the Amazon

### 5.1. Types and Sources of Agro-Extractivism Residues

**Figure 6:**
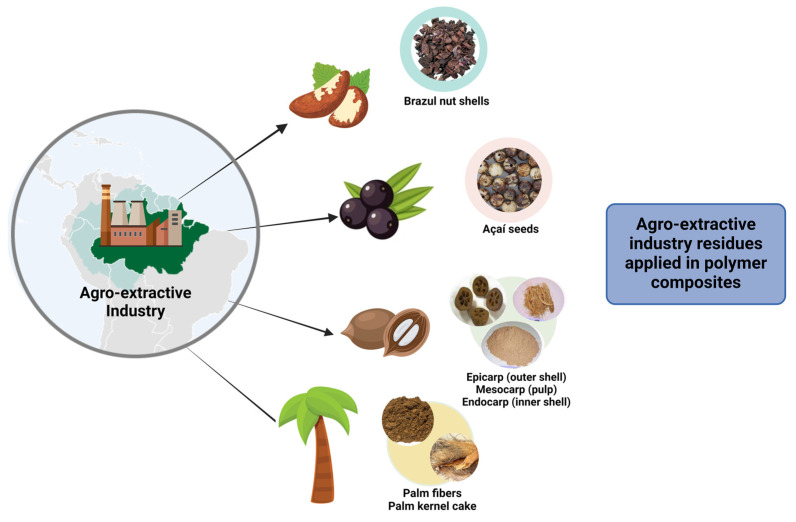
Agro-extractivism industry residues applied in polymer composites: Examples include Brazil nut shells, açaí seeds, babassu coconut components (epicarp, mesocarp, and endocarp), palm fibers, and palm kernel cake.

The Amazon region produces an extensive array of agro-extractivism residues, each with distinct characteristics that make them valuable for enhancing PCs. These residues are by-products of the processing of various Amazonian plants and include a wide variety of materials, such as seeds, shells, leaves, and bagasse [186]. These materials, which are often overlooked or underutilized, represent a significant untapped resource that can be harnessed to improve PCs’ properties. Figure 6 illustrates the application of residues from the Amazonian agro-extractivism industry in polymer composites, highlighting the diversity of materials available for this purpose. Brazil nut shells; açaí seeds; and the epicarp, mesocarp, and endocarp of babassu coconut, as well as palm fibers and palm kernel cake, are examples of traditionally underutilized by-products that present new opportunities for composite development. The integration of these residues into polymer matrices not only adds value to agro-industrial by-products but also contributes to sustainability and the circular economy in the AR. By utilizing these abundant renewable materials, it is possible to create composites with enhanced properties for various industrial applications.

Each type of residue offers a unique combination of physical and chemical properties that can be strategically leveraged to enhance the mechanical strength, thermal stability, and biodegradability of composites [131,187]. For example, seeds and shells, typically rich in lignin and cellulose, can provide structural reinforcement when incorporated into polymer matrices, resulting in composites with superior durability and strength. Leaves and bagasse, which are often fibrous and contain bioactive compounds, can improve the flexibility, impact resistance, and environmental sustainability of the composites [74,188,189]. A notable example is the Brazil nut shell (*Bertholletia excelsa*). This material has excellent mechanical strength and thermal stability, making it ideal for use in construction materials and automotive parts. Research has shown that the thick mesocarp of the Brazil nut, composed of fibers and sclereids, provides high compressive strength and toughness [190]. Additionally, the mesocarp’s unique microstructural properties, including a high lignin content, contribute to its ability to withstand high mechanical forces, further highlighting its potential in creating durable and impact-resistant materials [90]. Another valuable by-product is the açaí seed from the *Euterpe* palm. Often discarded during the processing of açaí fruits, these seeds are rich in lignin and cellulose, making them suitable for reinforcement in biodegradable polymers. The incorporation of açaí seed residues in PCs can improve mechanical properties and biodegradability, contributing to waste reduction and adding value to the agro-extractivism industry [191].

Tucumã seeds, which come from the fruit of the *Astrocaryum aculeatum* palm, are rich in fatty acids and antioxidants. These seeds can improve the thermal and mechanical properties of PCs [192,193]. Additionally, corn fiber, derived from harvest residues, can be used in biocomposite films. The addition of corn fiber improves the tensile strength and Young’s modulus of the films, as well as reducing moisture adsorption, making them suitable for biodegradable packaging [194]. Likewise, coir fiber, derived from the husk of coconuts, is a lignocellulosic material known for its high lignin content and durability. When incorporated into polymer matrices, coir fiber enhances mechanical properties such as tensile strength and thermal stability [195]. In a similar vein, palm kernel cake, a by-product of the palm oil industry, serves as an effective filler in polymer composites. Its lignocellulosic composition enhances tensile and flexural strength, especially when treated to improve bonding within the composite matrix [196].

### 5.2. Residue Availability and Environmental Impact in the Agro-Extractivism Industry

The açaí industry is a significant contributor to the agro-extractivism sector in the AR. In 2022, the processing of açaí fruits primarily for pulp and juice generated a substantial number of seed residues, which are estimated to constitute approximately 70% of the fruit’s total weight. This translates to more than 1.19 million tons of açaí seed waste being produced [62,197]. These residues, often underutilized, represent a significant biomass resource with potential applications in various industries. The vast availability of açaí seeds offers an excellent opportunity for their incorporation into PCs, which can enhance the mechanical properties of these materials and contribute to more sustainable manufacturing practices. By valorizing these residues, the açaí industry can mitigate environmental impacts, reduce waste, and promote a circular economy within the region [198,199].

The Brazil nut (Amazon nut) industry also produces a considerable amount of waste. Brazil’s annual production of Brazil nuts is estimated to be around 40.3 thousand tons. For every ton of cleaned nuts, approximately 1.4 tons of residues, including shells and the fruit’s outer casing (known as the ouriço), is generated. Consequently, the total amount of shells and ouriços produced exceeds 56 thousand tons per year [200,201]. This high generation of residues is due to the fruit being enclosed within the pericarp (ouriço) and the disposal of the mesocarp (shell), as well as broken nuts that do not meet commercial standards, as noted by Santos [201] in his research. This abundance of Brazil nut residues offers a valuable opportunity for sustainable material applications. Their properties make them ideal for incorporation into PCs, as they can reduce environmental impact, lower waste management costs, and contribute to eco-friendly composite development.

Another residue comes from the African oil palm (*Elaeis guineensis*, Jacq.), which was introduced into Brazil for oil extraction. The processing of oil palm fruits generates a variety of products and by-products, including crude palm oil, palm kernel oil, palm kernel cake, empty fruit bunches, press fiber, shells, and significant amounts of liquid effluent. As highlighted by Chavalparit et al. [202], for every ton of fresh fruit bunches processed, approximately 23% are empty fruit bunches, 14% are fiber, 5.5% are shells, 3.2% are decanter cake, and 5% are boiler ash. Based on these percentages, the annual production of 2.8 million tons of fresh fruit bunches in the Eastern Amazon results in approximately 640,000 tons of empty fruit bunches, 390,000 tons of fiber, 150,000 tons of shells, 90,000 tons of decanter cake, and 140,000 tons of boiler ash [62,202]. These residues, when incorporated into polymeric composites, reduce environmental impact, lower waste costs, and promote a circular economy, adding value to the oil palm industry.

The AR is rich in oil-producing industries, such as buriti, andiroba, and copaiba, which generate residues that can be effectively utilized in polymer composites, adding value to what would otherwise be waste. A prime example of this is the babassu coconut industry. After oil extraction, the remaining 93% of the fruit comprises 13% epicarp (rich in fibers), 20% mesocarp (high in starch), and 60% endocarp [203]. In 2022, babassu nut extraction in Brazil reached 30,478 tons, resulting in approximately 3963 tons of epicarp, 6096 tons of mesocarp, and 18,287 tons of endocarp residues [62]. Babassu residues constitute a significant biomass resource with diverse application potential. Despite their abundance, improper disposal has led to environmental concerns [172].

### 5.3. Applications of Agro-Extractivism Industry Residues in Polymer Composites

Researchers across the globe are exploring innovative ways to incorporate these materials into polymeric matrices, aiming to create sustainable and high-performance composites. In this context, several case studies have emerged, highlighting the practical applications and benefits of these composites in various industries. Table 4 presents a selection of examples from the literature, illustrating the improvements in mechanical properties, such as increased tensile strength and modulus, as well as reduced water absorption. These examples represent broader findings in the field, demonstrating the significant potential of agricultural and forest residues for enhancing composite performance.

One such example is the study by Kieling et al. [204], which investigated a novel wood–plastic composite made from recycled PP and Tucumã endocarp powder (TEP), addressing both social and environmental issues. The study highlights the significant potential of using TEP, a lignocellulosic residue abundantly produced in Manaus, Brazil, to enhance the properties of wood–plastic composites. Composites were produced with varying TEP content (10, 20, 30, 40, and 50 wt.%) through injection molding and were characterized using techniques such as SEM, FTIR, and mechanical testing. The incorporation of TEP increased the elastic modulus by up to 28% (from 0.73 GPa to 0.94 GPa) and the dynamic friction coefficient but reduced tensile strength by 59% (from 23.06 MPa to 9.36 MPa) and impact resistance by 76% (from 149 J/m to 35 J/m) as TEP content increased to 50 wt%. Despite these reductions in strength, the composites exhibited minimal water absorption (up to 1.6%) and maintained physical integrity after aging, making the wood–plastic composites promising candidates for sustainable engineering applications. The study demonstrates the feasibility of producing wood–plastic composites without coupling agents, utilizing waste materials that would otherwise contribute to environmental pollution. Composites with 20 wt.% TEP showed an optimal balance of mechanical properties, suggesting their potential for eco-friendly engineering solutions.

Barbosa et al. [191] investigated the use of açaí seed residue as a reinforcement material in PCs, motivated by the substantial amount of agro-waste generated from açaí processing. The study focused on two main factors: the particle size (granulometry) of the açaí seed residues and the percentage of resin used in the composite formulations. The results indicated that particle size significantly influenced water absorption and thickness swelling, with larger particles exhibiting lower water absorption rates. For example, the composite with larger particles and higher resin content showed the lowest water absorption after 24 h at 21.11%, compared to 56.65% for the composite with smaller particles and lower resin content. Additionally, the mechanical properties, such as screw withdrawal and internal bonding, were assessed. Although the screw withdrawal results did not meet the NBR 14810–2:2018 standard, the internal bonding test showed that composites with larger particle sizes performed better, with the largest particle size composite exhibiting the highest internal bonding strength. This suggests that larger particle sizes and lower resin weight fractions enhance bonding performance. The results highlighted the potential of açaí seed residues in creating sustainable composites, particularly for non-structural indoor applications like partitions and ceilings.

Palm kernel cake, a by-product of the palm oil extraction industry, has shown significant potential as a filler material in polymer composites. Although underutilized in the AR, Cionita et al. [196] demonstrated its effectiveness in reinforcing epoxy composites. The study incorporated PKC filler into epoxy resin at varying concentrations, with the best mechanical properties achieved at 30 wt.% filler loading. At this concentration, the composite exhibited a tensile strength of 31.20 MPa and a flexural strength of 39.70 MPa. When the filler loading increased to 40 wt.%, both the tensile and flexural strengths decreased due to weaker interfacial bonding, reaching values of 22.90 MPa and 30.50 MPa, respectively. Additionally, a 5 wt.% alkaline treatment using NaOH further enhanced the mechanical properties, improving the tensile strength by 23% and the tensile modulus by 15%, demonstrating that palm kernel cake, widely available in the Amazon due to the palm oil industry, could be a valuable and sustainable reinforcement material for polymer composites, supporting its broader adoption in the region.

## 6. Conclusions

This perspective highlights the immense potential of agro-extractivism and timber industry residues from the Amazon in the development of sustainable and high-performance polymer composites. Integrating these natural residues into polymer matrices can enhance both the mechanical and thermal properties, but more sustainable practices that contribute to the circular economy in the region can also be promoted. Composites made from residues like açaí seeds, Brazil nut shells, and wood sawdust demonstrate significant potential in improving tensile strength, thermal stability, and biodegradability. These materials provide a viable solution for waste management in the Amazon, transforming previously underutilized by-products into valuable resources for the industry.

Nevertheless, challenges remain, such as enhancing matrix-reinforcement compatibility and developing more efficient, low-impact manufacturing processes. Continued exploration in this area may address these issues by investigating new methods of chemical modification and the use of natural additives to further enhance composite properties. In summary, the incorporation of agro-extractivism and timber industry residues from the Amazon into polymer composites not only contributes to the valorization of these residues but also offers a promising pathway for developing more sustainable materials. This approach benefits local industries and supports global efforts toward sustainable and responsible development.

## Figures and Tables

**Table 1:** Chemical composition of Amazonian agro-industrial and wood residue.

Common Name (Scientific Name)	Cellulose (%)	Hemicellulose (%)	Lignin (%)	Extractives (%)	Ash (%)	Ref.
Açaí seeds (*Euterpe oleracea*)	11.58	37.88	14.12	4.09	1.57	[78]
Açaí seeds (*Euterpe oleracea*)	8.5	48.1	16.4	13.1	0.96	[79]
Palm kernel shell	29.7	47.7	53.4	-	1.1	[85]
Palm kernel shell	6.92	26.11	53.85	-	8.68	[86]
Maçaranduba sawdust (*Manilkara huberi*)	69.41 *	-	34.68	7.36	0.33	[89]
Brazil nut mesocarp (*Bertholletia excelsa*)	15.9	15.7	56	2.5	6.2	[90]
Brazil nut waste (*Bertholletia excelsa*)	-	37.09	55.76	4.54	2.61	[91]
Cupuaçu husk (*Theobroma grandiflorum*)	49.43	10.13	11.36	13.94	2.36	[92]
Maçaranduba sawdust (*Manilkara huberi*)	66 *	-	29	5	0	[93]
Mandioqueira wood (*Ruizterania albiflora*)	55.9	9.23	28.71	3.29	1.12	[94]
Cambará wood (*Vochysia* sp.)	49.5	12.56	32.28	0.18	3.9	[94]
Amescla wood (*Trattinnickia* sp.)	45.18	13.38	33.86	0.71	1.72	[94]
Angelim-pedra (*Hymenolobium petraeum*)	73.15 *	-	23.84	3.01	-	[95]

* Represents holocellulose, which is the sum of cellulose and hemicellulose.

**Table 2:** Overview of key characteristics of conventional and biodegradable polymers.

Polymer Type	Polymer	Mechanical Properties (Tensile Strength)	Manufacturing Efficiency	Environmental Impact	Ref.
Conventional	Polyethylene (PE)	(LDPE: 7–15 MPa HDPE: 31–42 MPa)	High efficiency, low cost	High impact, non-biodegradable, moderate recyclability	[106,111,133,134]
	Polypropylene (PP)	(3.45–30 MPa)	Moderate efficiency, low cost	High impact, high toxicity, non-biodegradable	[106,111,135,136]
	Polystyrene (PS)	(35.9–56.5 MPa)	Moderate efficiency, low cost	High impact, non-biodegradable non-recyclable	[137,138,139]
	Polyurethane (PU)	(31–62 MPa)	Moderate efficiency, moderate cost	High Impact, non-biodegradable, moderate recyclability	[137,140,141]
	Polyvinyl chloride (PVC)	(30–65 MPa)	Moderate efficiency, low cost	High impact, High Toxicity, Non-biodegradable, pollutant emissions	[137,142,143,144]
	Epoxy resins *	(60–115 MPa)	Low efficiency, low cost	High impact, non-biodegradable, non-recyclable	[145,146,147,148,149,150]
Biodegradable	Polybutylene succinate (PBS)	(18–25) MPa	Moderate efficiency, high cost	Low impact, biodegradable non-toxic by-products	[151,152,153,154]
	Polyhydroxyalkanoates (PHAs)	(20–40) MPa	Moderate efficiency, high cost	Low impact, biodegradable	[151,155,156,157]
	Polybutylene Adipate Terephthalate (PBAT)	(30–36) MPa	Moderate efficiency, high cost	Low impact, biodegradable, non-toxic by-products	[157,158,159,160]
	Polylactic acid (PLA)	(50–70 MPa)	Moderate efficiency, moderate cost	Low impact, biodegradable	[151,161,162,163]

* Different types of epoxy resins.

**Table 3:** Comparative analysis of mechanical properties of polymer composites reinforced with timber-industry residues.

Timber Residue	Composition	Improved Properties	Original Values	Improvement (%)	Ref.
Louro Itaúba, Louro Gamela, Maçaranduba	5–10% NaOH-treated wood residue	Rupture modulus: Louro Gamela (16 MPa), Louro Itaúba (9 MPa), Maçaranduba (11 MPa)	Louro Gamela (12 MPa), Louro Itaúba (2 MPa), Maçaranduba (3 MPa)	Gamela: +33%, Itaúba: +350%, Maçaranduba: +266%	[179]
Caryocar villosum, Hymenolobium excelsum, Tachigali myrmecophyla	8% Phenol-formaldehyde resin with wood residues	Rupture modulus (10.04 MPa), Young’s modulus (1616.74 MPa)	Rupture modulus: 8.5 MPa, Young’s modulus: 1400 MPa	Rupture: +18%, Elasticity: +15%	[180]
Maçaranduba, Pinus, sugarcane bagasse	PLA with 20% Maçaranduba/ Pinus residues	Young’s modulus: Pinus (+34%), Maçaranduba (+15%). Impact absorption: Maçaranduba (+0.357 J/m)	Young’s modulus: 2.6 GPa, impact absorption: 0.193 J/m	+34% (Pinus), +15% (Maçaranduba)	[183]
Jatobá + malva fibers	75% Jatobá wood + 25% malva fibers	Tensile strength: 26.06 MPa	Tensile strength: 25.09 MPa	+4%	[184]
Jatobá wood powder	40% Jatobá-wood powder in PP	Young’s modulus: 2000 MPa	Young’s modulus: 800 MPa	+59%	[185]

**Table 4:** Comparative analysis of mechanical properties of polymer composites reinforced with agro-extractivism industry residues.

Timber Residue	Composition	Improved Properties	Original Values	Improvement (%)	Ref.
Açaí seed	Polymer composite with 30% açaí seed	Lower water absorption (21.11%) with larger particles, better bonding strength	Water absorption: 56.65%	−63% water absorption	[191]
Palm kernel cake	Epoxy with 30% palm kernel cake	Tensile strength: 31.20 MPa, Flexural strength: 39.70 MPa	Tensile strength: 22.90 MPa, Flexural strength: 30.50 MPa	Tensile: +36%, Flexural: +30%	[196]
Tucumã endocarp	Recycled PP with 0–50 wt.% Tucumã endocarp powder (TEP)	Increased tensile and flexural modulus (+28% to +30%), improved compressive strength (+134%) with 40 wt.% TEP	Tensile modulus: 0.73 GPa (PP100) Flexural modulus: 1.13 GPa (PP100)	Modulus: +28% (0.94 GPa with 50 wt.% TEP) Flexural modulus: +30% (1.48 GPa with 50 wt.% TEP)	[204]
Açaí seed	Natural rubber with 0–50 phr açaí seed	Increased tensile strength, increased Young’s modulus	Tensile strength: 5.2 MPa (0 phr) Young’s modulus: 0.8 MPa (0 phr)	Tensile strength: +65% (at 50 phr) Young’s modulus: +127.5% (at 50 phr, 1.82 MPa)	[202]
Açaí seed fiber	PBAT/PLA (50/50 wt.%) with 30% açaí seed fiber	Increased elongation at break (+17%)	Elongation: 12.8% (PBAT/PLA blend)	Elongation: +17% (15.02%)	[205]
Babassu Mesocarp	PBAT/PHB (25/75, 50/50, 75/25) with 20% Babassu	Increased Young’s modulus (+19.4%), slight increase in stiffness	Young’s modulus: 334 MPa (50/50 blend)	Young’s modulus: +19.4% (for 25/75 blend with Babassu)	[206]
Buriti fiber	Epoxy (DGEBA/TETA) with 0%, 10%, 20%, 30% buriti fiber	Increased tensile strength (+22.8%), improved modulus (+57%) with 30% fiber content	Tensile strength: 61.94 MPa (0% fiber) Modulus: 0.97 GPa (0% fiber)	Tensile strength: +22.8% (76.07 MPa with 30% fiber) Modulus: +57% (1.52 GPa with 30% fiber)	[207]

## Data Availability

Not applicable.

## References

[1] Nepstad D., Azevedo-Ramos C., Lima E., Mcgrath D., Pereira C., Merry F. (2004). Managing the Amazon Timber Industry. Conserv. Biol..

[2] Zaman K. (2022). Environmental cost of deforestation in Brazil’s Amazon Rainforest: Controlling biocapacity deficit and renewable wastes for conserving forest resources. For. Ecol. Manag..

[3] Azevedo F.P.M., Oliveira M.A.d., Rocha L.L., Cardoso A., Veroneze G.d.M. (2022). The circular economy in the perspective of sustainable joinery: A case study in the Amazon/A economia circular na perspectiva da carpintaria sustentável: Um estudo de caso na Amazônia. Braz. J. Dev..

[4] Väisänen T., Haapala A., Lappalainen R., Tomppo L. (2016). Utilization of agricultural and forest industry waste and residues in natural fiber-polymer composites: A review. Waste Manag..

[5] Das O., Babu K., Shanmugam V., Sykam K., Tebyetekerwa M., Neisiany R.E., Försth M., Sas G., Gonzalez-Libreros J., Capezza A.J. (2022). Natural and industrial wastes for sustainable and renewable polymer composites. Renew. Sustain. Energy Rev..

[6] Shanmugam V., Mensah R.A., Försth M., Sas G., Restás Á., Addy C., Xu Q., Jiang L., Neisiany R.E., Singha S. (2021). Circular economy in biocomposite development: State-of-the-art, challenges and emerging trends. Compos. Part C Open Access.

[7] Lopes E., Soares-Filho B., Souza F., Rajão R., Merry F., Carvalho Ribeiro S. (2019). Mapping the socio-ecology of Non-Timber Forest Products (NTFP) extraction in the Brazilian Amazon: The case of açaí (Euterpe precatoria Mart) in Acre. Landsc. Urban Plan..

[8] Ribeiro M.B.N., Jerozolimski A., de Robert P., Magnusson W.E. (2014). Brazil nut stock and harvesting at different spatial scales in southeastern Amazonia. For. Ecol. Manag..

[9] Andrade F.W.C., Pinto T.I., Moreira L.d.S., da Ponte M.J.M., Lobato T.d.C., de Sousa J.T.R., Moutinho V.H.P. (2022). The Legal Roundwood Market in the Amazon and Its Impact on Deforestation in the Region between 2009–2015. Forests.

[10] Satyanarayana K.G., Arizaga G.G., Wypych F. (2009). Biodegradable composites based on lignocellulosic fibers—An overview. Prog. Polym. Sci..

[11] Samaniego-Aguilar K., Sánchez-Safont E., Rodríguez A., Marín A., Candal M.V., Cabedo L., Gamez-Perez J. (2023). Valorization of Agricultural Waste Lignocellulosic Fibers for Poly(3-Hydroxybutyrate-Co-Valerate)-Based Composites in Short Shelf-Life Applications. Polymers.

[12] Platnieks O., Barkane A., Ijudina N., Gaidukova G., Thakur V.K., Gaidukovs S. (2020). Sustainable tetra pak recycled cellulose/Poly (Butylene succinate) based woody-like composites for a circular economy. J. Clean. Prod..

[13] Vallejos M.E., Felissia F.E., Area M.C., Ehman N.V., Tarrés Q., Mutjé P. (2016). Nanofibrillated cellulose (CNF) from eucalyptus sawdust as a dry strength agent of unrefined eucalyptus handsheets. Carbohydr. Polym..

[14] Shinoj S., Visvanathan R., Panigrahi S., Kochubabu M. (2011). Oil palm fiber (OPF) and its composites: A review. Ind. Crops Prod..

[15] Fávaro S.L., Lopes M.S., Vieira de Carvalho Neto A.G., Rogério de Santana R., Radovanovic E. (2010). Chemical, morphological, and mechanical analysis of rice husk/post-consumer polyethylene composites. Compos. Part A Appl. Sci. Manuf..

[16] Low J.H., Andenan N., Rahman W.A.W.A., Rusman R., Majid R.A. (2017). Evaluation of rice straw as natural filler for injection molded high density polyethylene bio-composite materials. Chem. Eng. Trans..

[17] Singh Y., Singh J., Sharma S., Lam T.D., Nguyen D.N. (2020). Fabrication and characterization of coir/carbon-fiber reinforced epoxy-based hybrid composite for helmet shells and sports-good applications: Influence of fiber surface modifications on the mechanical, thermal and morphological properties. J. Mater. Res. Technol..

[18] Monteiro S.N., Candido V.S., Braga F.O., Bolzan L.T., Weber R.P., Drelich J.W. (2016). Sugarcane bagasse waste in composites for multilayered armor. Eur. Polym. J..

[19] Ghaffar S.H., Fan M. (2017). An aggregated understanding of physicochemical properties and surface functionalities of wheat straw node and internode. Ind. Crops Prod..

[20] Branciforti M.C., Marinelli A.L., Kobayashi M., Ambrosio J.D., Monteiro M.R., Nobre A.D. (2009). Wood Polymer Composites Technology Supporting the Recovery and Protection of Tropical Forests: The Amazonian Phoenix Project. Sustainability.

[21] Marques M., Nascimento C.C.d., Araujo R.D.d. (2020). Ecological panels as an alternative for waste from mechanical processing of Amazonian species. Int. J. Innov. Educ. Res..

[22] Thoppul S.D., Finegan J., Gibson R.F. (2009). Mechanics of mechanically fastened joints in polymer–matrix composite structures—A review. Compos. Sci. Technol..

[23] Dang Z.M., Yuan J.K., Zha J.W., Zhou T., Li S.T., Hu G.H. (2012). Fundamentals, processes and applications of high-permittivity polymer–matrix composites. Prog. Mater. Sci..

[24] U.S. Congress, Office of Technology Assessment (1988). Advanced Materials by Design.

[25] Rajak D.K., Pagar D.D., Kumar R., Pruncu C.I. (2019). Recent progress of reinforcement materials: A comprehensive overview of composite materials. J. Mater. Res. Technol..

[26] Huang S., Fu Q., Yan L., Kasal B. (2021). Characterization of interfacial properties between fibre and polymer matrix in composite materials—A critical review. J. Mater. Res. Technol..

[27] Rahman M.A., Haque S., Athikesavan M.M., Kamaludeen M.B. (2023). A review of environmentally friendly green composites: Production methods, current progresses, and challenges. Environ. Sci. Pollut. Res..

[28] Taurino R., Bondioli F., Messori M. (2023). Use of different kinds of waste in the construction of new polymer composites: Review. Mater. Today Mater. Today Sustain..

[29] Todorov S.D., Pieri F.A. (2017). Tropical Fruits: From Cultivation to Consumption and Health Benefits, Fruits from the Amazon.

[30] Almeida N.A. (2016). Óleos essenciais e desenvolvimento sustentável na Amazônia: Uma aplicação da matriz de importância e desempenho. Reflexões Econômicas.

[31] da S. (2011). Santos, A. Óleos Essenciais: Uma Abordagem Econômica e Industrial.

[32] Galúcio A.V., Prudente A.L. (2019). Museu Goeldi: 150 Anos de Ciências na Amazônia.

[33] Mafra R.Z., Medeiros R.L. (2017). Estudos da Bioindústria Amazonense: Sustentabilidade, Mercado e Tecnologia.

[34] dos Santos J.D., Medeiros R.L., Kuwahara N., Pauly P.R., Pierre Filho M.d.Q., Ferreira M.A.C., da Rocha S.D., da Costa E.B.S. (2023). Selection of suppliers in a bioindustry in the Amazon using the saw, topsis and promethee II methods combined with fuzzy logic. Rev. Gestão Secr..

[35] Oliveira R., Lasmar D., Mafra R., Kieling A., Albuquerque A., Oliveira L., Oliveira S. (2023). Mapeamento das empresas e institutos de pesquisas que utilizam a biotecnologia Industrial no Estado do Amazonas. Peer Rev..

[36] Souza N.S. (2010). Boletim Científico ESMPU. Bol. Científico ESMPU.

[37] Guayasamin J.M., Ribas C.C., Carnaval A.C., Carrillo J.D., Hoorn C., Lohmann L.G., Riff D., Ulloa Ulloa C., Albert J.S. (2024). Evolution of Amazonian biodiversity: A review. Acta Amaz..

[38] Brandão D.O. (2023). Desmatamento na Amazônia e Influência Nos Produtos Florestais Não-Madeireiros de Uso Econômico Local. Doctor’s Thesis.

[39] Rocha M.I., Benkendorf S., Gern R.M.M., Riani J.C., Wisbeck E. (2020). Desenvolvimento de biocompósitos fúngicos utilizando resíduos industriais. Matéria.

[40] de Sousa Matos T., de Sousa de Matos T., da Silva A.C.R., Monteiro S.N., Candido V.S. Obtenção e Caracterização de Biocompósitos de Resíduo de Amido Mandioca Reforçados com Fibra de Tururi. Proceedings of the 76° Congresso Anual da ABM—Internacional.

[41] de Araújo Ribeiro K.C., da Silva C.A., Freire L.H.C. (2019). Ecodesign via Biocompósitos Poliméricos: Envelhecimento, ANÁLISE Estrutural e Reciclagem. Mix Sustentável.

[42] de Aragão D.I., Cardoso P.H.M., Lima E.M.B., da S. M. (2016). Thiré, R.M. Caracterização de biocompósitos de poli(3-hidroxibutirato-co-3-hidroxivalerato) (PHBV)/coproduto da agroindústria do suco de manga. J. Agric. Food Ind. Technol..

[43] Meazza K. (2019). Avaliação do Beneficiamento de Resíduos do Pirarucu Para a Produção de Biocompósitos a Base de Hidroxiapatita e Fibra de Colágeno. Doctor’s Thesis.

[44] Vinod U., Sanjay M., Siengchin S., Parameswaranpillai J. (2020). A comprehensive review on the sustainable approach for recycling of textiles and apparel for production of biocomposites. J. Clean. Prod..

[45] Manu T., Nazmi A.R., Shahri B., Emerson N., Huber T. (2022). Mechanical and morphological properties of banana fiber reinforced polylactic acid biocomposites. Mater. Today Mater. Today Commun..

[46] Rodrigues G.d.M., Filgueiras C.T., Garcia V.A.d.S., Carvalho R.A.d., Velasco J.I., Fakhouri F.M. (2020). Antimicrobial activity and GC-MS profile of copaiba oil for incorporation in Xanthosoma mafaffa Schott starch-based films. Polymers.

[47] Silva T.R.d., Matos P.R.d., Tambara Júnior L.U.D., Marvila M.T., Azevedo A.R.G.d. (2021). Development of a biomimetic material for wound healing using poly(e-caprolactone) and andiroba seed oil. J. Build. Eng..

[48] Santos R.P.d.O., Castro D.O., Ruvolo-Filho A.C., Frollini E. (2014). Preparation and properties of curaua fiber-reinforced polypropylene composites. J. Appl. Polym. Sci..

[49] Reddy N., Jiang J., Yang Y. (2014). Biodegradable composites containing chicken feathers as matrix and jute fibers as reinforcement. J. Polym. Environ..

[50] Costa U.O., Nascimento L.F.C., Garcia J.M., Bezerra W.B.A., Monteiro S.N. (2020). Development and characterization of wood-plastic composite produced with high wood residue content. J. Mater. Res. Technol..

[51] Rojas-Bringas P.M., De-la-Torre G.E., Torres F.G. (2021). Influence of the Source of Starch and Plasticizers on the Environmental Burden of Starch-Brazil Nut Fiber Biocomposite Production: A Life Cycle Assessment Approach. Sci. Total Environ..

[52] Inamura P.Y., Shimazaki K., Colombo M.A., Rosa R., Moura E.A.B., del Mastro N.L. (2010). Effect of Electron Beam Irradiation on Mechanical Properties of Gelatin/Brazil Nut Shell Fiber Composites. Matéria.

[53] Souza L., Souza L., Silva F. (2017). Autogenous self-healing capacity of curauá fiber-reinforced cement biocomposite. Procedia Eng..

[54] Castro D., Ruvolo-Filho A., Frollini E. (2012). Preparation and characterization of a biocomposite using hydroxylated polybutadiene, high-density bio-polyethylene and curauá fiber. Polym. Test..

[55] Frollini E., Bartolucci N., Sisti L., Celli A. (2015). Mechanical properties, water absorption, and morphology of a biocomposite using poly (butylene succinate) (PBS) and curauá fiber. Polym. Test..

[56] Andrade M., dos Santos H., Nunes F., Costa J., Lentini M. (2022). Produção de Madeira e Diversidade de Espécies Arbóreas Exploradas na Amazônia Brasileira: Situação Atual e Recomendações para o Setor Florestal. https://oeco.org.br/wp-content/uploads/2022/08/Boletim_Timberflow_oito-2022-1.pdf.

[57] Dionisio L.F.S. (2020). Efeitos a médio prazo da exploração seletiva no crescimento, mortalidade e recrutamento de *Manilkara huberi* (Ducke) A. Chev. em uma floresta amazônica. Sci. For..

[58] Castro T.d.C., Carvalho J.O.P.d. (2014). Dinâmica da População de Manilkara huberi (Ducke) A. Chev. Durante 26 Anos após a Exploração Florestal em Uma Área de Terra Firme na Amazônia Brasileira. Ciência Florest..

[59] Melo R.R.d., Dacroce J.M.F., Rodolfo Junior F., Lisboa G.d.S., França L.C.d.J. (2019). Lumber Yield of Four Native Forest Species of the Amazon Region. Floresta E Ambiente.

[60] Siliprandi N.C., Nogueira E.M., Toledo J.J., Fearnside P.M., Nascimento H.E.M. (2016). Inter-site variation in allometry and wood density of *Goupia glabra* Aubl. in Amazonia. Braz. J. Biol..

[61] IBAMA (2024). Report on Timber Industry Waste in the Amazon.

[62] IBGE Systematic Survey of Agricultural Production. https://sidra.ibge.gov.br.

[63] Figueira J., Almeida M., Silva R. (2020). Waste Production in Amazonian Sawmills. J. Environ. Manag..

[64] Muñiz-Miret N., Vamos R., Hiraoka M., Montagnini F., Mendelsohn R.O. (1996). The economic value of managing the açaí palm (Euterpe oleracea Mart.) in the floodplains of the Amazon estuary, Pará, Brazil. For. Ecol. Manag..

[65] Kainer K.A., Wadt L.H.O., Staudhammer C.L. (2018). The evolving role of Bertholletia excelsa in Amazonia: Contributing to local livelihoods and forest conservation. Desenvolv. E Meio Ambiente.

[66] Santos A., Guerra F. (2010). Aspectos econômicos da cadeia produtiva dos óleos de andiroba (Carapa guianensis Aubl.) e copaíba (Copaifera multijuga Hayne) na Floresta Nacional do Tapajós—Pará. Floresta.

[67] Ferreira S.F., Buller L.S., Maciel-Silva F.W., Sganzerla W.G., Berni M.D., Forster-Carneiro T. (2021). Waste management and bioenergy recovery from açaí processing in the Brazilian Amazonian region: A perspective for a circular economy. Biofuels Bioprod. Biorefining.

[68] da Silva A.S.O., de Campos Paraense V. (2019). Production chain for brazil-nuts (Bertholletia excelsa Bonpl.) at Ipaú-Anilzinho extractive reserve, municipality of Baião, Pará, Amazonian Brazil. Rev. Agro@Mbiente-Line.

[69] International Nut and Dried Fruit Council (INC) (2023). INC Nuts & Dried Fruits Statistical Yearbook 2022/23. https://inc.nutfruit.org/wp-content/uploads/2023/05/Statistical-Yearbook-2022-2023.pdf.

[70] Mafra R.Z., Lasmar D.J., Vilela D.C. (2019). Relacionamentos Interorganizacionais na Bioindústria Amazonense na Percepção dos Empresários. Rev. De Adm. Contemp..

[71] Machado M., Rocha A., Tartarotti N. (2023). Sustainable Development as a Wicked Problem: The Case of the Brazilian Amazon Region. Eur. J. Sustain. Dev..

[72] Rao J., Lv Z., Chen G., Peng F. (2023). Hemicellulose: Structure, chemical modification, and application. Prog. Polym. Sci..

[73] Abolore R.S., Jaiswal S., Jaiswal A.K. (2024). Green and sustainable pretreatment methods for cellulose extraction from lignocellulosic biomass and its applications: A review. Carbohydr. Polym. Technol. Appl..

[74] Zhang C.W., Nair S.S., Chen H., Yan N., Farnood R., Li F.Y. (2020). Thermally stable, enhanced water barrier, high strength starch bio-composite reinforced with lignin containing cellulose nanofibrils. Carbohydr. Polym..

[75] Zhang C., Xue J., Yang X., Ke Y., Ou R., Wang Y., Madbouly S.A., Wang Q. (2022). From plant phenols to novel bio-based polymers. Prog. Polym. Sci..

[76] Belgacem M.N., Gandini A. (2005). The surface modification of cellulose fibres for use as reinforcing elements in composite materials. Compos. Interfaces.

[77] Colson J., Pettersson T., Asaadi S., Sixta H., Nypelö T., Mautner A., Konnerth J. (2018). Adhesion properties of regenerated lignocellulosic fibres towards poly (lactic acid) microspheres assessed by colloidal probe technique. J. Colloid Interface Sci..

[78] Murillo-Franco S.L., Galvis-Nieto J.D., Orrego C.E. (2023). Physicochemical characterization of açaí seeds (Euterpe oleracea) from Colombian pacific and their potential of mannan-oligosaccharides and sugar production via enzymatic hydrolysis. Biomass Convers. Biorefin..

[79] Buratto R.T., Cocero M.J., Martín Á. (2021). Characterization of industrial açaí pulp residues and valorization by microwave-assisted extraction. Chem. Eng. Process. Process Intensif..

[80] Monteiro A.F., Miguez I.S., Silva J.P.R.B., Silva A.S. (2019). High concentration and yield production of mannose from açaí (Euterpe oleracea Mart.) seeds via mannanase-catalyzed hydrolysis. Sci. Rep..

[81] Rambo M.K.D., Schmidt F.L., Ferreira M.M.C. (2015). Analysis of the lignocellulosic components of biomass residues for biorefinery opportunities. Talanta.

[82] Millane R.P., Hendrixson T.L. (1994). Crystal structures of mannan and glucomannans. Carbohydr. Polym..

[83] Várdai R., Schäffer Á., Ferdinánd M., Lummerstorfer T., Jerabek M., Gahleitner M., Faludi G., Móczó J., Pukánszky B. (2022). Crystalline structure and reinforcement in hybrid PP composites. J. Therm. Anal. Calorim..

[84] Fuadi N.A., Ibrahim A.S., Ismail K.N. Review study for activated carbon from palm shell used for treatment of waste water. Proceedings of the Conference on Waste Water Treatment.

[85] Okoroigwe E.C., Saffron C.M., Kamdem P.D. (2014). Characterization of palm kernel shell for materials reinforcement and water treatment. Chem. Eng. J. Chem. Eng..

[86] Zhao J., Duan K., Zhang J.W., Lu X., Weng J. (2010). The influence of polymer concentrations on the structure and mechanical properties of porous polycaprolactone-coated hydroxyapatite scaffolds. Appl. Surf. Sci..

[87] Costa W.A., Bezerra F.W.F., Oliveira M.S., Andrade E.H.A., Santos A.P.M., Cunha V.M.B., Santos D.C.S., Banna D.A.D.S., Teixeira E., Carvalho Junior R.N. (2019). Supercritical CO_2_ extraction and transesterification of the residual oil from industrial palm kernel cake with supercritical methanol. J. Supercrit. Fluids.

[88] Dominici F., Samper M.D., Carbonell-Verdu A., Luzi F., López-Martínez J., Torre L., Puglia D. (2020). Improved toughness in lignin/natural fiber composites plasticized with epoxidized and maleinized linseed oils. Materials.

[89] Nobre J., Napoli A., Bianchi M., Silva M., Numazawa S. Use of residues of Manilkara huberi from the state of Pará in the production of activated carbon. Proceedings of the XIV EBRAMEM.

[90] Sonego M., Fleck C., Pessan L.A. (2019). Mesocarp of Brazil nut (Bertholletia excelsa) as inspiration for new impact resistant materials. Bioinspir. Biomim..

[91] Leandro R.I.M., Abreu J.J.C., Martins C.S., Santos I.S., Bianchi M.L., Nobre J.R.C. (2019). Elementary, Chemical and Energy Characteristics of Brazil Nuts Waste (Bertholletia excelsa) in the State of Pará. Floresta Ambiente.

[92] Marasca N., Brito M.R., Rambo M.C.D., Pedrazzi C., Scapin E., Rambo M.K.D. (2022). Analysis of the potential of cupuaçu husks (Theobroma grandiflorum) as raw material for the synthesis of bioproducts and energy generation. Food Sci. Technol..

[93] Medeiros D.T. (2020). Avaliação de Propriedades Tecnológicas da Madeira de Quatro Espécies da Amazônia. Bachelor’s Thesis.

[94] Bimestre T.A., Silva F.S., Tuna C.E., dos Santos J.C., de Carvalho J.A., Canettieri E.V. (2023). Physicochemical Characterization and Thermal Behavior of Different Wood Species from the Amazon Biome. Energies.

[95] Almeida A.P.S., Rodrigues D.A., Castelo P.A.R. (2014). Determination of Chemical Properties of Woods from Southern Amazon. Sci. Electron. Arch..

[96] Brahmakumar M., Pavithran C., Pillai R.M. (2005). Coconut fibre reinforced polyethylene composites: Effect of natural waxy surface layer of the fibre on fibre/matrix interfacial bonding and strength of composites. Compos. Sci. Technol..

[97] Yue H., Zheng Y., Zheng P., Guo J., Fernández-Blázquez J.P., Clark J.H., Cui Y. (2020). On the improvement of properties of bioplastic composites derived from wasted cottonseed protein by rational cross-linking and natural fiber reinforcement. Green Chem..

[98] La Mantia F., Morreale M. (2011). Green composites: A brief review. Compos. Part A Appl. Sci. Manuf..

[99] Maraveas C. (2020). Production of Sustainable and Biodegradable Polymers from Agricultural Waste. Polymers.

[100] Hsissou R., Seghiri R., Benzekri Z., Hilali M., Rafik M., Elharfi A. (2021). Polymer composite materials: A comprehensive review. Compos. Struct..

[101] Khatami M., Bairwan R., Khalil H. (2024). The Role of Natural Fiber Reinforcement in Thermoplastic Elastomers Biocomposites. Fibers Polym..

[102] Wu Klingler W., Bifulco A., Polisi C., Huang Z., Gaan S. (2023). Recyclable inherently flame-retardant thermosets: Chemistry, properties and applications. Compos. Part B Eng..

[103] Song J.H., Murphy R.J., Narayan R., Davies G.B.H. (2009). Biodegradable and compostable alternatives to conventional plastics. Philos. Trans. R. Soc. B Biol. Sci..

[104] Samir A., Ashour F., Hakim A. (2022). Recent advances in biodegradable polymers for sustainable applications. NPJ Mater. Degrad..

[105] Gopanna A., Rajan K.P., Thomas S.P., Chavali M., Grumezescu V., Grumezescu A.M. (2019). Chapter 6—Polyethylene and polypropylene matrix composites for biomedical applications. Materials for Biomedical Engineering.

[106] Potrykus M., Redko V., Głowacka K., Piotrowicz-Cieślak A., Szarlej P., Janik H., Wolska L. (2021). Polypropylene structure alterations after 5 years of natural degradation in a waste landfill. Sci. Total Environ..

[107] Alsabri A., Tahir F., Al-Ghamdi S.G. (2021). Life-Cycle Assessment of Polypropylene Production in the Gulf Cooperation Council (GCC) Region. Polymers.

[108] Danyluk C., Erickson R., Burrows S., Auras R. (2010). Industrial Composting of Poly (Lactic Acid) Bottles. J. Test. Eval..

[109] Taylor R., Nattrass L., Alberts G., Robson P., Chudziak C., Bauen A., Libelli I.M., Lotti G., Prussi M., Nistri R. (2015). From the Sugar Platform to Biofuels and Biochemicals: Final Report for the European Commission Directorate-General Energy.

[110] Khan R.A., Khan M.A., Sultana S., Nuruzzaman Khan M., Shubhra Q.T.H., Noor F.G. (2010). Mechanical, Degradation, and Interfacial Properties of Synthetic Degradable Fiber Reinforced Polypropylene Composites. J. Reinf. Plast. Compos..

[111] Yin S., Tuladhar R., Shanks R.A., Collister T., Combe M., Jacob M., Tian M., Sivakugan N. (2015). Fiber preparation and mechanical properties of recycled polypropylene for reinforcing concrete. J. Appl. Polym. Sci..

[112] Gigante V., Canesi I., Cinelli P., Coltelli M.B., Lazzeri A. (2019). Rubber Toughening of Polylactic Acid (PLA) with Poly (butylene adipate-co-terephthalate) (PBAT): Mechanical Properties, Fracture Mechanics and Analysis of Ductile-to-Brittle Behavior while Varying Temperature and Test Speed. Eur. Polym. J..

[113] Tsou C.-H., Chen Z.-J., Yuan S., Ma Z.-L., Wu C.-S., Yang T., Jia C.-F., De Guzman M.R. (2022). The preparation and performance of poly (butylene adipate) terephthalate/corn stalk composites. Curr. Res. Green Sustain. Chem..

[114] Bourban P.E., Bögli A., Bonjour F., Månson J.-A.E. (1998). Integrated processing of thermoplastic composites. Compos. Sci. Technol..

[115] Chan C.M., Vandi L.J., Pratt S., Halley P., Richardson D., Werker A., Laycock B. (2018). Composites of Wood and Biodegradable Thermoplastics: A Review. Polym. Rev..

[116] Arif Z.U., Khalid M.Y., Sheikh M.F., Zolfagharian A., Bodaghi M. (2022). Biopolymeric sustainable materials and their emerging applications. J. Environ. Chem. Eng..

[117] Pedras X., Piñeiro G., Castilla M. (2020). Application of agroforestry residues in polymeric composite materials. Mater. Compuestos.

[118] Oladele I.O., Okoro C.J., Taiwo A.S., Onuh L.N., Agbeboh N.I., Balogun O.P., Olubambi P.A., Lephuthing S.S. (2023). Modern Trends in Recycling Waste Thermoplastics and Their Prospective Applications: A Review. J. Compos. Sci..

[119] Vaidya U.K., Chawla K.K. (2008). Processing of fibre reinforced thermoplastic composites. Int. Mater. Rev..

[120] Norizan M.N., Rahmah M. (2017). A Review: Fibres, Polymer Matrices and Composites. Pertanika J. Sci. Technol..

[121] Campos R.D., Sotenko M., Hosur M., Jeelani S., Díaz F.R.V., Moura E.A.B., Kirwan K. (2015). Effect of Mercerization and Electron-Beam Irradiation on Mechanical Properties of High-Density Polyethylene (HDPE)/Brazil Nut Pod Fiber (BNPF) Bio-Composites. Charact. Miner. Met. Mater..

[122] Souza P., Rodrigues E., Prêta J., Goulart S., Mulinari D. (2011). Mechanical properties of HDPE/textile fibers composites. Procedia Eng..

[123] Gomes D.A.C., de Novais Miranda E.H., de Araújo Veloso M.C.R., da Silva M.G., Ferreira G.C., Mendes L.M., Júnior J.B.G. (2023). Production and characterization of recycled low-density polyethylene/amazon palm fiber composites. Ind. Crops Prod..

[124] Ortega F., Versino F., López O. (2022). Biobased composites from agro-industrial wastes and by-products. Emergent Mater..

[125] Yadav R., Singh M., Shekhawat D., Lee S.Y., Park S.J. (2023). The role of fillers to enhance the mechanical, thermal, and wear characteristics of polymer composite materials: A review. Compos. Part A Appl. Sci. Manuf..

[126] Zhao X., Long Y., Xu S., Liu X., Chen L., Wang Y.Z. (2023). Recovery of epoxy thermosets and their composites. Mater. Today.

[127] Mendes C.P., Fleming R., Goncalves A.M.B., da Silva M.J., Prataviera R., Cena C. (2021). Mechanical and microstructural characterization of epoxy/sawdust (*Pinus elliottii*) composites. Polym. Compos. Polym. Compos..

[128] Rajeshkumar G., Arvindh Seshadri S., Devnani G., Sanjay M., Siengchin S., Prakash Maran J., Al-Dhabi N.A., Karuppiah P., Mariadhas V.A., Sivarajasekar N. (2021). Environment friendly, renewable and sustainable poly lactic acid (PLA) based natural fiber reinforced composites—A comprehensive review. J. Clean. Prod..

[129] Moshood T.D., Nawanir G., Mahmud F., Mohamad F., Ahmad M.H., AbdulGhani A. (2022). Sustainability of biodegradable plastics: New problem or solution to solve the global plastic pollution?. Curr. Res. Green Sustain. Chem..

[130] Scaffaro R., Maio A., Sutera F., Gulino E.F., Morreale M. (2019). Degradation and Recycling of Films Based on Biodegradable Polymers: A Short Review. Polymers.

[131] Ferreira F.V., Pinheiro I.F., Mariano M., Cividanes L.S., Costa J.C., Nascimento N.R., Kimura S.P., Neto J.C., Lona L.M. (2019). Environmentally friendly polymer composites based on PBAT reinforced with natural fibers from the amazon forest. Polym. Compos..

[132] Pinheiro I., Morales A., Mei L. (2014). Polymeric biocomposites of poly (butylene adipate-co-terephthalate) reinforced with natural Munguba fibers. Cellulose.

[133] Zhong X., Zhao X., Qian Y., Zou Y. (2018). Polyethylene plastic production process. Insight Mater. Sci..

[134] Dem K. Polyethylene vs. Polypropylene: When to Choose What?. https://omnexus.specialchem.com/tech-library/article/polyethylene-versus-polypropylene.

[135] Shubhra Q., Alam A.K.M.M., Quaiyyum M.A. (2011). Mechanical properties of polypropylene composites: A review. J. Thermoplast. Compos. Mater..

[136] Alsabri A., Tahir F., Al-Ghamdi S.G. (2022). Environmental impacts of polypropylene (PP) production and prospects of its recycling in the GCC region. Mater. Today Proc..

[137] Cambridge University Engineering Department (2003). Materials Data Book.

[138] Samper M.D., Garcia-Sanoguera D., Parres F., López J. (2010). Recycling of Expanded Polystyrene from Packaging. Prog. Rubber Plast. Recycl. Technol..

[139] Samaras N.N.T., Perry E. (1951). Commercial production of polystyrene. J. Appl. Chem..

[140] Cregut M., Bedas M., Durand M.-J., Thouand G. (2013). New insights into polyurethane biodegradation and realistic prospects for the development of a sustainable waste recycling process. Biotechnol. Adv..

[141] Zhang Y., Wang J., Fang X., Liao J., Zhou X., Zhou S., Bai F., Peng S. (2019). High solid content production of environmentally benign ultra-thin lignin-based polyurethane films: Plasticization and degradation. Polymer.

[142] Comaniţă E.-D., Ghinea C., Roşca M., Simion I.M., Petraru M., Gavrilescu M. Environmental impacts of polyvinyl chloride (PVC) production process. Proceedings of the 2015 E-Health and Bioengineering Conference.

[143] Rahmatabadi D., Soltanmohammadi K., Aberoumand M., Soleyman E., Ghasemi I., Baniassadi M., Abrinia K., Bodaghi M., Baghani M. (2023). Development of Pure Poly Vinyl Chloride (PVC) with Excellent 3D Printability and Macro- and Micro-Structural Properties. Macromol. Mater. Eng..

[144] Akovali G., Pacheco-Torgal F., Jalali S., Fucic A. (2012). Plastic materials: Polyvinyl chloride (PVC). Toxicity of Building Materials.

[145] Yang J.-P., Chen Z.-K., Yang G., Fu S.-Y., Ye L. (2008). Simultaneous improvements in the cryogenic tensile strength, ductility and impact strength of epoxy resins by a hyperbranched polymer. Polymer.

[146] Engelberg P.I., Tesoro G.C. (1990). Mechanical and thermal properties of epoxy resins with reversible crosslinks. Polym. Eng. Sci..

[147] Luo T., Zhao Y., Fu K., Cui X., Chen B. (2024). High-efficiency manufacturing of epoxy resins through two-point initiation of frontal polymerization. Chem. Eng. J..

[148] Jin F.-L., Li X., Park S.-J. (2015). Synthesis and application of epoxy resins: A review. J. Ind. Eng. Chem..

[149] Eliaz N., Ron E.Z., Gozin M., Younger S., Biran D., Tal N. (2018). Microbial Degradation of Epoxy. Materials.

[150] Luoma G.A., Rowland R.D. (1986). Environmental degradation of an epoxy resin matrix. J. Appl. Polym. Sci..

[151] Changwichan K., Silalertruksa T., Gheewala S.H. (2018). Eco-Efficiency Assessment of Bioplastics Production Systems and End-of-Life Options. Sustainability.

[152] Saeed U., Taimoor A.A., Rather S., Al-Zaitone B., Al-Turaif H. (2023). Characterization of cellulose nanofibril reinforced polybutylene succinate biocomposite. J. Thermoplast. Compos. Mater..

[153] Saffian H.A., Yamaguchi M., Ariffin H., Abdan K., Kassim N.K., Lee S.H., Lee C.H., Shafi A.R., Alias A.H. (2021). Thermal, Physical and Mechanical Properties of Poly (Butylene Succinate)/Kenaf Core Fibers Composites Reinforced with Esterified Lignin. Polymers.

[154] Aliotta L., Seggiani M., Lazzeri A., Gigante V., Cinelli P. (2022). A Brief Review of Poly (Butylene Succinate) (PBS) and Its Main Copolymers: Synthesis, Blends, Composites, Biodegradability, and Applications. Polymers.

[155] Ivanov V., Stabnikov V., Ahmed Z., Dobrenko S., Saliuk A. (2015). Production and Applications of Crude Polyhydroxyalkanoate-Containing Bioplastic from the Organic Fraction of Municipal Solid Waste. Int. J. Environ. Sci. Technol..

[156] Saratale R.G., Cho S.-K., Saratale G.D., Kadam A.A., Ghodake G.S., Kumar M., Bharagava R.N., Kumar G., Kim D.S., Mulla S.I. (2021). A comprehensive overview and recent advances on polyhydroxyalkanoates (PHA) production using various organic waste streams. Bioresour. Technol..

[157] Ten E., Jiang L., Zhang J., Wolcott M.P., Misra M., Pandey J.K., Mohanty A.K. (2015). Mechanical performance of polyhydroxyalkanoate (PHA)-based biocomposites. Biocomposites.

[158] BASF ecoflex® F Blend C1200. https://www.Plasticsportal.Net/Wa/PlasticsEU~en_GB/Function/Conversions:/Publish/Common/Upload/Biodegradable_plastics/Ecoflex_F_Blend_C1200.Pdf.

[159] Bai J., Pei H., Zhou X., Xie X. (2021). Reactive compatibilization and properties of low-cost and high-performance PBAT/thermoplastic starch blends. Eur. Polym. J..

[160] Souza P.M.S., Sommaggio L.R.D., Marin-Morales M.A., Morales A.R. (2020). PBAT biodegradable mulch films: Study of ecotoxicological impacts using Allium cepa, Lactuca sativa and HepG2/C3A cell culture. Chemosphere.

[161] Azlin M.N.M., Sapuan S.M., Zuhri M.Y.M., Zainudin E.S. (2022). Mechanical, Morphological and Thermal Properties of Woven Polyester Fiber Reinforced Polylactic Acid (PLA) Composites. Fibers Polym..

[162] Jonoobi M., Harun J., Mathew A.P., Oksman K. (2010). Mechanical properties of cellulose nanofiber (CNF) reinforced polylactic acid (PLA) prepared by twin screw extrusion. Compos. Sci. Technol..

[163] Yu J.-M., Xu S., Liu B., Wang H., Qiao F., Ren X., Wei Q. (2023). PLA bioplastic production: From monomer to the polymer. Eur. Polym. J..

[164] Campos E., Punhagui K., John V. (2021). CO_2_ footprint of Amazon lumber: A meta-analysis. Resour. Conserv. Recycl..

[165] Richardson V., Peres C. (2016). Temporal Decay in Timber Species Composition and Value in Amazonian Logging Concessions. PLoS ONE.

[166] Morais P., Reis A., Moraes M., Christoforo A., Nascimento M., Lahr F., Silva M. (2024). Determinação do processo produtivo de painéis homogêneos de partículas com resíduos amazônicos com identificação anatômica da biomassa. Cad. Pedagógico.

[167] Ramos W., Ruivo M., Jardim M., Sousa L. (2018). Geração de resíduos madeireiros do setor de base florestal na região metropolitana de Belém, Pará. Ciência Florest..

[168] Mendoza Z.M.d.S.H.d., Evangelista W.V., Araújo S.d.O., Souza C.C.d., Ribeiro F.D.L., Silva J.d.C. (2010). Análise dos resíduos madeireiros gerados nas marcenarias do município de Viço—Minas Gerais. Rev. Árvore.

[169] Grantham J., Ellis T. (1974). Potentials of Wood for Producing Energy. J. For..

[170] Manhães J. (1994). Uso da Biomassa Florestal como Fonte de Energia. Floresta Ambiente.

[171] Sommerhuber P., Welling J., Krause A. (2015). Substitution potentials of recycled HDPE and wood particles from post-consumer packaging waste in Wood–Plastic Composites. Waste Manag..

[172] Dias J.M.C.d.S., dos Santos D.T., Braga M., Onoyama M.M., Miranda C.H.B., Barbosa P.F.D., Rocha J.D. (2012). Produção de Briquetes e Péletes a Partir de Resíduos Agrícolas, Agroindustriais E Florestais.

[173] Cárdenas-Zapata R., Palma-Ramírez D., Flores-Vela A., Romero-Partida J., Paredes-Rojas J., Márquez-Rocha F., Bravo-Díaz B. (2022). Structural and thermal study of hemicellulose and lignin removal from two types of sawdust to isolate cellulose. MRS Adv..

[174] Venkatarajan S., Subbu C., Athijayamani A., Muthuraja R. (2021). Mechanical properties of natural cellulose fibers reinforced polymer composites—2015–2020: A review. Mater. Today Proc..

[175] Tuoto M. (2009). Levantamento sobre a geração de resíduos provenientes da atividade madeireira e proposição de diretrizes para políticas, normas e condutas técnicas para promover o seu uso adequado. Ministério Meio Ambiente Curitiba.

[176] da Silva Luz E., Soares A.A.V., Goulart S.L., Carvalho A.G., Monteiro T.C., de Paula Protásio T. (2021). Challenges of the lumber production in the Amazon region: Relation between sustainability of sawmills, process yield and logs quality. Environ. Dev. Sustain..

[177] United Nations (2016). UN Comtrade Database. https://comtradeplus.un.org.

[178] Phiri R., Mavinkere Rangappa S., Siengchin S., Oladijo O.P., Dhakal H.N. (2023). Development of sustainable biopolymer-based composites for lightweight applications from agricultural waste biomass: A review. Adv. Ind. Eng. Polym. Res..

[179] dos Santos G.M., de Araújo A.B.S., Giacon V.M., Faez R. (2023). Does the moisture content of mercerized wood influence the modulus of rupture of thermopressed polyurethane-based composites?. Ind. Crops Prod..

[180] Surdi P.G., Bortoletto Júnior G., Castro V.R.d., Brito F.M.S., Berger M.d.S., Zanuncio J.C. (2019). Particleboard Production with Residues from Mechanical Processing of Amazonian Woods. Rev. Árvore.

[181] Ramli R.A. (2024). A comprehensive review on utilization of waste materials in wood plastic composite. Mater. Today Mater. Today Sustain..

[182] Cintra S.C., Braga N.F., Morgado G.F.d.M., Montanheiro T.L.d.A., Marini J., Passador F.R., Montagna L.S. (2021). Development of new biodegradable composites materials from polycaprolactone and wood flour. Wood Mater. Sci. Eng..

[183] Rocha D.B., de Souza A.G., Szostak M., Rosa D.d.S. (2020). Polylactic acid/Lignocellulosic residue composites compatibilized through a starch coating. Polym. Compos..

[184] da Costa D.S., Banna W.R.E., Fujiyama R.T. (2023). Wood Residue of Jatobá (Hymenaea courbaril) and Short Fiber of Malva in Composites. Rev. De Gestão Soc. E Ambient..

[185] Ferreira E.S.B., Luna C.B.B., Araújo E.M., Siqueira D.D., Wellen R.M.R. (2019). Polypropylene/wood powder composites: Evaluation of PP viscosity in thermal, mechanical, thermomechanical, and morphological characters. J. Thermoplast. Compos. Mater..

[186] Ferreira-Leitão V., Gottschalk L.M.F., Ferrara M.A., Nepomuceno A.L., Molinari H.B.C., Bon E.P.S. (2010). Biomass Residues in Brazil: Availability and Potential Uses. Waste Biomass Valorization.

[187] Sun J., Pang Y., Yang Y., Zhao J., Xia R., Li Y., Liu Y., Guo H. (2019). Improvement of Rice Husk/HDPE Bio-Composites Interfacial Properties by Silane Coupling Agent and Compatibilizer Complementary Modification. Polymers.

[188] Lala S.D., Deb P., Barua E., Deoghare A.B., Chatterjee S. (2023). A comparative study on mechanical, physico-chemical, and thermal properties of rubber and walnut seed shell reinforced epoxy composites and prominent timber species. Proc. Inst. Mech. Eng. Part C J. Mech. Eng. Sci..

[189] Naduparambath S., Sreejith M.P., Jinitha T.V., Shaniba V., Aparna K.B., Purushothaman E. (2018). Development of green composites of poly (vinyl alcohol) reinforced with microcrystalline cellulose derived from sago seed shells. Polym. Compos..

[190] Soares C., Moura E., Arenhardt V., Deliza E.E.V., Pedro Filho F.d.S. (2023). Biotechnology management in the Amazon and the production of polypropylene/Brazil Nut Shell fiber biocomposite. Rev. Gestão Secr..

[191] de Melo Barbosa A., dos Santos G.M., de Melo G.M.M., Litaiff H.A., Martorano L.G., Giacon V.M. (2022). Evaluation of the use of açaí seed residue as reinforcement in polymeric composite. Polym. Compos. Polym. Compos..

[192] Rayssa S., Lima A.P.A.d.C., Conte-Junior C.A. (2023). Health from Brazilian Amazon food wastes: Bioactive compounds, antioxidants, antimicrobials, and potentials against cancer and oral diseases. Crit. Rev. Food Sci. Nutr..

[193] Fonseca A.S., Raabe J., Dias L.M., Baliza A.E.R., Costa T.G., Silva L.E., Vasconcelos R.P., Marconcini J.M., Savastano H., Mendes L.M. (2019). Main Characteristics of Underexploited Amazonian Palm Fibers for Using as Potential Reinforcing Materials. Waste Biomass Valorization.

[194] Lenhani G.C., dos Santos D.F., Koester D.L., Biduski B., Deon V.G., Machado Junior M., Pinto V.Z. (2021). Application of Corn Fibers from Harvest Residues in Biocomposite Films. J. Polym. Environ..

[195] Adeniyi A.G., Onifade D.V., Ighalo J.O., Adeoye A.S. (2019). A review of coir fiber reinforced polymer composites. Compos. Part B Eng..

[196] Cionita T., Siregar J.P., Shing W.L., Hee C.W., Fitriyana D.F., Jaafar J., Junid R., Irawan A.P., Hadi A.E. (2022). The Influence of Filler Loading and Alkaline Treatment on the Mechanical Properties of Palm Kernel Cake Filler Reinforced Epoxy Composites. Polymers.

[197] Lemos C., Cardoso F., Neumann F., Felix J., Teixeira L. Valorização de Resíduos da Indústria do Açaí: Oportunidades e Desafios. Report Prepared by the SENAI Institute for Innovation in Biosynthetics and Fibers.

[198] Araújo S., Santos G., Tolosa G., Hiranobe C., Budemberg E., Cabrera F., Silva M., Paim L., Job A., dos Santos R. (2023). Acai Residue as an Ecologic Filler to Reinforcement of Natural Rubber Biocomposites. Mater. Res..

[199] Silva T.O.M. (2017). Compostos voláteis, perfil de aroma, de ácidos graxos e potencial antioxidante de óleo extraído por prensagem a frio de resíduo agroindustrial de açaí (*Euterpe oleracea* Mart.). Master’s Thesis.

[200] Santos J.P., Braga L.F., Ruedell C.M., Seben Júnior G.d.F., Ferbonink G.F., Caione G. (2018). Caracterização física de substratos contendo resíduos de cascas de amêndoas de castanha-do-brasil (Bertholletia excelsa H.B.K.). Rev. Ciências Ambient..

[201] Santos M.J.T.d. (2014). Aproveitamento de Resíduos da Indústria de óleos Vegetais Produzidos na Amazônia. Master’s Thesis.

[202] Chavalparit O., Rulkens W.H., Mol A.P.J., Khaodhair S. (2006). Options for Environmental Sustainability of the Crude Palm Oil Industry in Thailand Through Enhancement of Industrial Ecosystems. Environ. Dev. Sustain..

[203] Machado N.A.F., Andrade H.A.F.d., Parra-Serrano L.J., Furtado M.B., Silva-Matos R.R.S.d., Farias M.F.d., Furtado J.d.L.B. (2017). Technological Characterization and Use of Babassu Residue (Orbygnia phalerata Mart.) in Particleboard. J. Agric. Sci..

[204] Kieling A.C., Santana G.P., Santos M.C.D., Neto J.C.D.M., Pino G.G.D., Santos M.D.D., Duvoisin S., Panzera T.H. (2021). Wood-plastic Composite Based on Recycled Polypropylene and Amazonian Tucumã (*Astrocaryum aculeatum*) Endocarp Waste. Fibers Polym..

[205] Wataya C.H., Lima R.A., Oliveira R.R., Moura E.A.B., Carpenter J.S., Bai C., Escobedo J.P., Hwang J.-Y., Ikhmayies S., Li B., Li J., Monteiro S.N., Peng Z., Zhang M. (2016). Mechanical, Morphological and Thermal Properties of Açaí Fibers Reinforced Biodegradable Polymer Composites. Characterization of Minerals, Metals, and Materials 2015.

[206] Beber V.C., De Barros S., Banea M.D., Brede M., De Carvalho L.H., Hoffmann R., Costa A.R.M., Bezerra E.B., Silva I.D.S., Haag K. (2018). Effect of Babassu Natural Filler on PBAT/PHB Biodegradable Blends: An Investigation of Thermal, Mechanical, and Morphological Behavior. Materials.

[207] Portela T.G.R., Costa L.L., Monteiro S.N. Resistência à Tração de Compósitos Poliméricos Reforçados com Fibras Alinhadas de Buriti. Proceedings of the 65° Congresso ABM.

